# Transcriptome Analysis of Porcine PBMCs Reveals the Immune Cascade Response and Gene Ontology Terms Related to Cell Death and Fibrosis in the Progression of Liver Failure

**DOI:** 10.1155/2018/2101906

**Published:** 2018-04-12

**Authors:** YiMin Zhang, Li Shao, Ning Zhou, JianZhou Li, Yu Chen, Juan Lu, Jie Wang, ErMei Chen, ZhongYang Xie, LanJuan Li

**Affiliations:** ^1^State Key Laboratory for Diagnosis and Treatment of Infectious Diseases, Collaborative Innovation Center for Diagnosis and Treatment of Infectious Diseases, The First Affiliated Hospital, College of Medicine, Zhejiang University, Hangzhou, Zhejiang Province, China; ^2^Department of Experimental Animals, Zhejiang Academy of Traditional Chinese Medicine, Hangzhou, Zhejiang Province, China

## Abstract

**Background:**

The key gene sets involved in the progression of acute liver failure (ALF), which has a high mortality rate, remain unclear. This study aims to gain a deeper understanding of the transcriptional response of peripheral blood mononuclear cells (PBMCs) following ALF.

**Methods:**

ALF was induced by D-galactosamine (D-gal) in a porcine model. PBMCs were separated at time zero (baseline group), 36 h (failure group), and 60 h (dying group) after D-gal injection. Transcriptional profiling was performed using RNA sequencing and analysed using DAVID bioinformatics resources.

**Results:**

Compared with the baseline group, 816 and 1,845 differentially expressed genes (DEGs) were identified in the failure and dying groups, respectively. A total of five and two gene ontology (GO) term clusters were enriched in 107 GO terms in the failure group and 154 GO terms in the dying group. These GO clusters were primarily immune-related, including genes regulating the inflammasome complex and toll-like receptor signalling pathways. Specifically, GO terms related to cell death, including apoptosis, pyroptosis, and autophagy, and those related to fibrosis, coagulation dysfunction, and hepatic encephalopathy were enriched. Seven Kyoto Encyclopedia of Genes and Genomes (KEGG) pathways, cytokine-cytokine receptor interaction, hematopoietic cell lineage, lysosome, rheumatoid arthritis, malaria, and phagosome and pertussis pathways were mapped for DEGs in the failure group. All of these seven KEGG pathways were involved in the 19 KEGG pathways mapped in the dying group.

**Conclusion:**

We found that the dramatic PBMC transcriptome changes triggered by ALF progression was predominantly related to immune responses. The enriched GO terms related to cell death, fibrosis, and so on, as indicated by PBMC transcriptome analysis, seem to be useful in elucidating potential key gene sets in the progression of ALF. A better understanding of these gene sets might be of preventive or therapeutic interest.

## 1. Introduction

Acute liver failure (ALF) is a severe syndrome characterised by hepatic encephalopathy and coagulation dysfunction, which can lead to multiorgan failure and death [1–3]. High morbidity and mortality following ALF are major problems worldwide [2, 3]. Thus, a thorough understanding of key genes or gene sets that regulate the progression of ALF is required.

The development of second-generation sequencing, particularly RNA-sequencing (RNA-Seq), has made it possible to perform global analysis of changes in gene expression during the course of a disease [4–6].

Taking biopsy samples during an ALF flare places the patient at high risk for lethal bleeding. More importantly, biopsy would influence the progression of ALF.

Analysis of the transcriptome of peripheral blood mononuclear cells (PBMCs) has successfully elucidated the mechanisms of numerous complex diseases and vaccination models [7–10]. These studies showed that analysing the PBMC transcriptome is helpful in identifying key genes and gene sets that control disease progression.

Here, we performed a comparative analysis of PBMC transcriptome in a porcine model of D-galactosamine- (D-gal-) induced ALF to identify candidate genes and gene sets that play important roles in the progression of ALF.

## 2. Materials and Methods

### 2.1. Porcine Model of D-gal-Induced ALF

A D-gal-induced ALF porcine model was used as previously described by our group [11]. Briefly, male Bama experimental miniature pigs were used and 1.3 g/kg body weight D-gal (Hanhong Chemical, Shanghai, China) was intravenously injected to induce ALF. Blood samples were collected at baseline (time zero) and 36 and 60 h after D-gal injection. Pigs were sacrificed after blood sample collection at 60 h. The general medical condition of the experimental pigs was monitored throughout the experiment.

All animal experiments were conducted in the Department of Experimental Animals, Zhejiang Academy of Traditional Chinese Medicine, China, and approved by the Animal Care Ethics Committee of the Academy. All experimental animals were treated humanely.

### 2.2. Clinical Parameters following D-gal-Induced Porcine ALF

At 0, 36, and 60 h, parameters to quantify the severity of liver failure were collected including the international normalization ratio (INR), and alanine aminotransferase, aspartate aminotransferase, alkaline phosphatase, *γ*-glutamyl transpeptidase, total bilirubin, and creatinine levels.

Blood ammonia was measured using an ammonia test kit (ARKRAY, Tokyo, Japan) with a detection range between 10 and 400 *μ*g/dL. INR was quantified using STA-R (Diagnostic Stago, Asnieres, France) in the emergency laboratory at the First Affiliated Hospital, College of Medicine, Zhejiang University. Serum alanine aminotransferase, aspartate aminotransferase, alkaline phosphatase, *γ*-glutamyl transpeptidase, total bilirubin, and creatinine levels were measured using an automated biochemical analyser (Abbott Aeroset; Abbott Laboratories, Chicago, IL, USA) in the same laboratory.

### 2.3. PBMC Isolation and RNA Extraction

PBMCs were isolated using Ficoll-Histopaque (Sigma Aldrich, St. Louis, MO, USA) immediately after blood sample collection. Subsequently, total RNA was extracted using RNeasy Mini kits (QIAGEN, Hilden, Germany) according to the manufacturer's instructions. All RNA samples were stored at −80°C for future analysis.

### 2.4. mRNA Library Construction, RNA-Sequencing, and Data Analysis

Total RNA (1 *μ*g) was thawed to create a library using TruSeq Stranded RNA LT Guide (Illumina, San Diego, CA, USA) according to the manufacturer's instructions. An Agilent 2100 bioanalyser (Santa Clara, CA, USA) was used to evaluate the concentration and size distribution of complementary DNA (cDNA) in the library before sequencing with the Illumina HiSequation 2500 system. The high-throughput sequencing was performed according to the manufacturer's instructions (Illumina HiSequation 2500 User Guide).

The raw data were filtered by FASTX (ver. 0.0.13) before mapping to the genome using TopHat (ver. 2.0.9). Gene fragments were counted using HTSeq followed by trimmed mean of *M* values (TMM) normalization. Significantly differentially expressed genes (DEGs) were identified using Cufflinks (ver. 2.2.1) [12]. DEGs were then submitted to Visualisation and Integrated Discovery analysis (DAVID; ver. 6.8) [13] for gene ontology (GO) term enrichment and clustering and Kyoto Encyclopedia of Genes and Genomes (KEGG) pathway mapping using default parameters, except for an EASE score setting of 0.05.

### 2.5. Validation of RNA-Seq Data by qRT-PCR

Quantitative RT-PCR was performed on selected genes to validate the data obtained from mRNA sequencing. Briefly, total RNA was reverse-transcribed into cDNA using the Fast Quant RT kit (Tiangen, Beijing, China). All qRT-PCR was conducted using SYBR Green SuperReal PreMix Plus (FP205; Tiangen) on an ABI 7900HT (Applied Biosystems, Foster City, CA, USA). Experimental conditions included a 3-min cycle at 94°C followed by 40 cycles of 20 s at 94°C, 20 s at 58°C, and 20 s at 72°C.

Each qRT-PCR run was performed in triplicate with two biological replicates. Beta-2-microglobulin (B2M) was used as the reference gene for data normalization, as previously described. A correlation analysis of the fold change of selected genes between qRT-PCR and RNA-Seq was performed.

### 2.6. Statistical Analysis

RNA-seq data analyses were described previously in Section 2.4. Other statistical analyses were performed by Graphpad Prism (Version 5.0, GraphPad Software, San Diego, United States). Biochemical parameters in the progress of ALF were compared using Student's *t*-test. Linear regression was performed in validation of RNA-Seq data by qRT-PCR. A *p* value less than 0.05 was considered significant.

## 3. Results

### 3.1. Clinical Features and Biochemical Parameters of D-gal-Induced ALF in Pigs

All animals enrolled in this experiment were healthy, with a good appetite and response to the D-gal injection at time zero (baseline). The ALF model was successfully established in all the animals at 36 h (failure) after D-gal injection. The pigs stopped eating and became obviously restless, with yellow urine. At 60 h post-injection (dying), the pigs showed ataxia and symptoms of hepatic encephalopathy, with no reaction to painful stimuli.

The biochemical parameters as ALF progressed are listed in Table 1. Liver failure was identified by the progressive increase in liver enzymes, bilirubin, blood ammonia, and the international normalization ratio in both the failure and dying groups as compared to the baseline group. A deviation of bilirubin and liver enzymes, or elevated total bilirubin with decreased liver enzymes, was observed in the dying group but not in the failure group.

### 3.2. Statistical Analysis of PBMC Transcriptome Data

RNA-Seq was performed in a total of 12 samples, with 4 samples in each group (baseline, failure, and dying). More than 9 Gb sequence data was the yield in each sample. Overall, 80.3–122.5 million raw reads per sample were generated with the quality of over 94.8%* Q*20, in which 66.3–118.0 million were clean reads.

A total of 54.1–83.5 million reads were mapped to the porcine genome, in which 63.4–83.4% fell in genic regions while the remaining were in intergenic regions. 14,990 to 15,812 expressed genes were identified (fragments per kilobase of exon per million mapped reads [FPKM] > 0) in each sample, respectively. Detailed information is presented in Table 2.

### 3.3. Differential Expression of Genes Associated with the Progression of D-gal-Induced ALF

**Figure 1:**
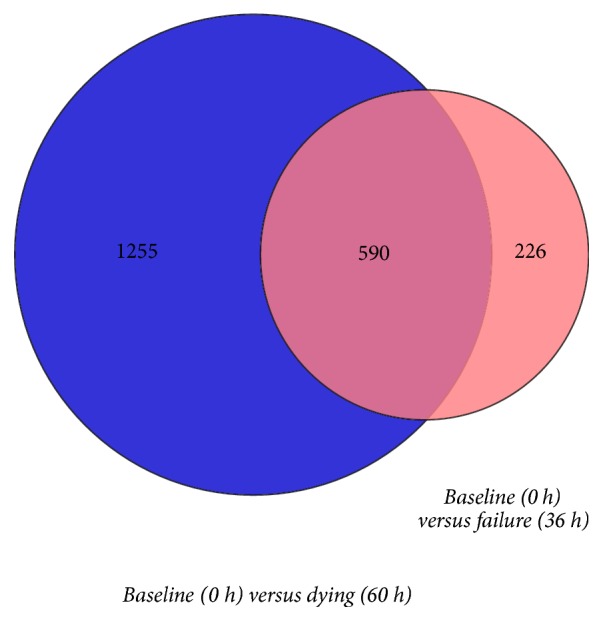
Differential expression of genes involved in the progression of acute liver failure (ALF).

DEGs during progression of D-gal-induced ALF were identified using Cufflinks (ver. 2.2.1). Genes were identified as significantly different with a false discovery rate (FDR) when the adjusted *p* value was (<0.05) and a greater than twofold log change was evident. Compared to the baseline group, 816 DEGs (Supplementary Table 1) were identified in the failure group and 1,845 DEGs (Supplementary Table 2) were identified in the dying group. A total of 590 identified genes overlapped between the two groups. Details are presented in Figure 1.

### 3.4. Progression of D-gal-Induced ALF: GO Analysis

GO enrichment analysis and term clustering were performed to identify DEGs in the failure and dying groups as compared to the baseline group. In total, 107 GO terms were enriched for DEGs identified in the failure group, of which 76 were within the biological process (BP) category, 15 were within the cellular component (CC) category, and 16 were within the molecular function (MF) category. Among these GO terms, 26 were grouped into five independent clusters. The GO terms in the five clusters were related to positive regulation of the inflammatory response, the inflammasome complex, the toll-like receptor (TLR) signalling pathway, cell chemotaxis, and semaphorin receptor activity. With the exception of predominantly innate immune-related terms, important GO terms related to cell death were also enriched for processes such as apoptosis and pyroptosis. GO terms related to autophagy, another type of programmed cell death, were also identified. These terms are the regulation of autophagy, phagocytic vesicles, and lysosomes. Terms related to the process of liver fibrosis included gene sets important in the negative regulation of the fibroblast growth factor receptor signalling pathway and semaphorin receptor activity, and so on. Moreover, GO terms related to coagulation dysfunction and hepatic encephalopathy were also enriched, such as blood coagulation, astrocyte development, and the semaphorin-plexin signalling pathway involved in axon guidance, branchiomotor neuron axon guidance, and so on.

In total, 154 GO terms were enriched for the DEGs identified in the dying group, which included 104 BP terms, 25 CC terms, and 25 MF terms. Overall, 20 out of 154 GO terms were included in two clusters. The representative GO terms in these clusters were related to regulation of the inflammatory response and the inflammasome complex. Most enriched GO terms were predominantly immune-related. Other important GO terms were related to cell death, including apoptotic processes, apoptotic-signalling pathways, negative regulation of apoptotic processes, pyroptosis, autophagy, lysosomal membrane, and lysosomal lumen. Fibrosis-related terms such as collagen catabolic processes and collagen binding were also enriched; hepatic encephalopathy-related GO term, astrocyte development, was also enriched.

**Figure 2:**
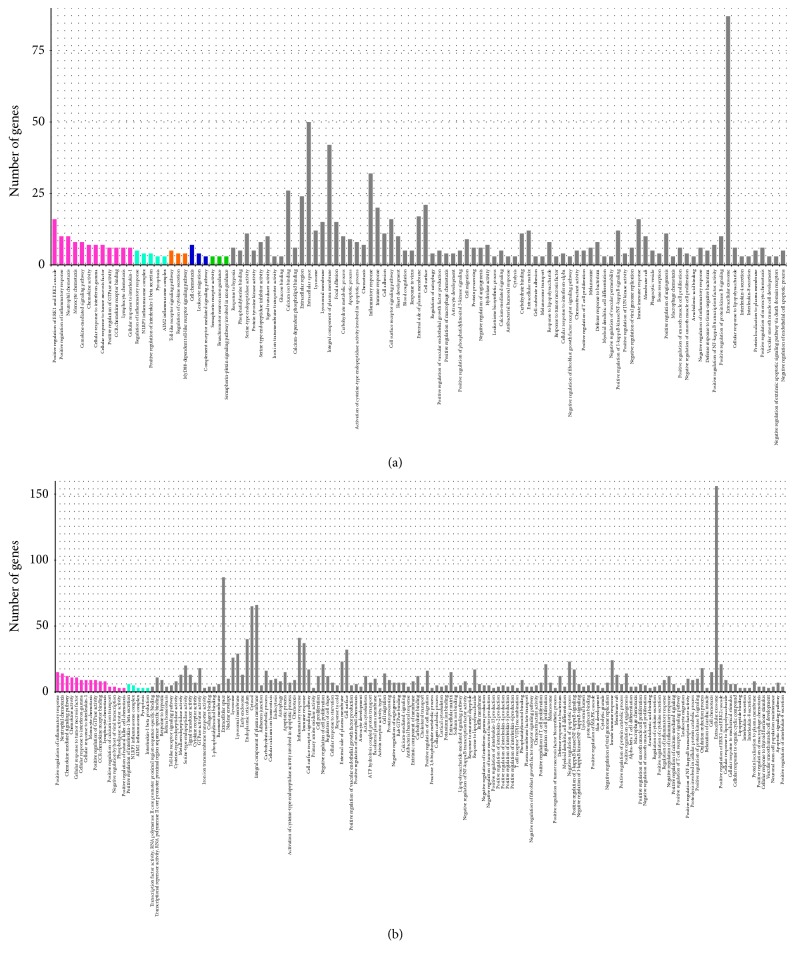
(a) Gene ontology (GO) terms of differentially expressed genes (DEGs) in the failure versus baseline group. (b) GO terms of DEGs in the dying versus baseline group.

Details of GO enrichment and clustering are presented in Figure 2.

### 3.5. KEGG Pathways Involved in the Progression of D-gal-Induced ALF

KEGG pathway mapping was used to study the molecular interactions and relation networks of the identified DEGs participating in metabolism, cellular processes and so on following D-gal-induced ALF. A total of seven KEGG pathways were mapped from DEGs identified in the failure group, all of which overlapped with the 19 identified KEGG pathways in the dying group. The seven KEGG pathways that were common to both included cytokine-cytokine receptor interaction, hematopoietic cell lineage, lysosome, rheumatoid arthritis, malaria, phagosome, and pertussis pathways. The remaining 12 KEGG pathways identified in the dying group were predominantly immune-related pathways, such as the NF-kappa B signalling pathway, the tumour necrosis factor (TNF) signalling pathway, and the complement and coagulation cascade pathways. KEGG pathways of diseases characterised by impaired liver function, such as Chagas disease (American trypanosomiasis),* Salmonella* infection, and Legionellosis, were also mapped using KEGG pathway mapping. Details are presented in Table 3.

### 3.6. Validation of RNA-Seq Data by qRT-PCR Analysis

**Figure 3:**
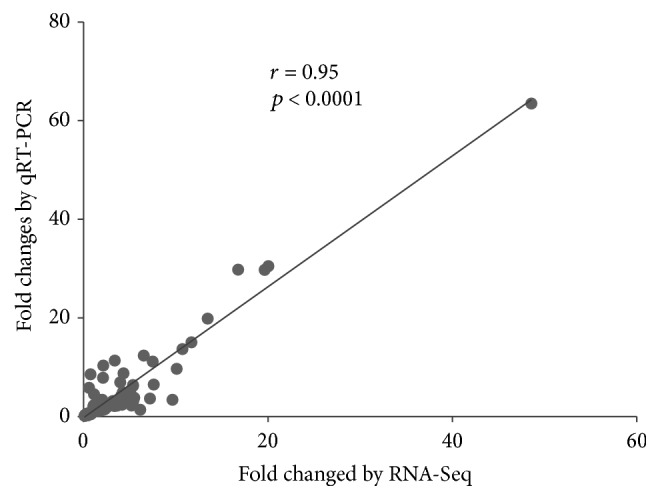
Correlation of gene fold changes between RNA-sequencing (RNA-Seq) and qRT-PCR analysis.

To validate the RNA-Seq data, qRT-PCR of 12 selected genes was performed. The forward and reverse pairs of qRT-PCR primers for each gene are listed in Supplementary Table 3. Linear correlation analysis was conducted between the RNA-Seq and qRT-PCR results, which showed that the fold changes were significantly concordant between RNA-Seq and qRT-PCR data (*r* = 0.95, *p* < 0.0001). Results are shown in Figure 3 and Supplementary Table 4.

## 4. Discussion

ALF is a syndrome characterised by severe coagulopathy due to liver dysfunction and altered consciousness as a result of hepatic encephalopathy [3]. These features of ALF can be revealed at the PBMC level by transcriptome analysis, with the enriched GO term of blood coagulation and the mapped KEGG pathway of complement and coagulation cascades. Vemuganti et al. reported that, in association with hepatic encephalopathy, axon guidance micro-RNA levels changed in the cerebral cortex of a rat model of ALF [14]. In this study, three GO terms—branchiomotor neuron axon guidance, the semaphorin-plexin signalling pathway involved in axon guidance, and the cortical cytoskeleton—were identified. KEGG mapping analysis also identified disease-related KEGG pathways characterised by liver dysfunction, such as malaria, Chagas disease (American trypanosomiasis),* Salmonella* infection, and Legionellosis. The ability of PBMCs to migrate in a transendothelial manner and establish a dialogue between cells in solid organs has been reported previously [15–17]. These findings may explain the transcriptome changes observed in PBMCs that parallel the changes observed in solid organs, such as the liver and brain.

Previous studies have revealed extensive differential gene expression detected in the liver during the progression of ALF [18]. In this study, compared to the baseline group, the number of DEGs identified in the dying group was more extensive than in the failure group (1845 and 816 genes, resp.), which suggests that the cascades identified by PBMC transcriptome analysis change as ALF progresses. In addition, seven common KEGG pathways were identified for DEGs in both the failure and dying groups, which showed that the key pathways triggered by ALF result in further cascades at the transcriptome level.

Systemic inflammatory responses play an important role in the progression of ALF. Several key innate and adaptive immune mechanisms of ALF have been described previously, including acquired neutrophil dysfunction [19, 20], TLR function [21–23], and the important actions of chemokine and cytokine storms [24, 25]. All of these immune-related changes were identified in our GO enrichment and KEGG pathway mapping studies, which included genes involved in neutrophil chemotaxis, the TLR signalling pathway, and the TNF signalling pathway, among others.

Cell death plays an important role in ALF [26, 27]. Apoptosis is a form of hepatocyte death that contributes to ALF [28, 29]. Evidence of apoptotic pathways was identified in our transcriptome analysis. Two GO terms, the apoptotic process and apoptotic-signalling pathways, were enriched. Apart from apoptosis, recent research has focused on a new form of proinflammatory cell death known as pyroptosis [30]. Until now, studies on pyroptosis in ALF have been limited [31, 32]. Furthermore, to the best of our knowledge, a role for pyroptosis in drug-induced ALF has not been reported previously. The enrichment of pyroptosis GO terms following D-gal-induced ALF suggests that pyroptosis is an important route to cell death in a model of drug-induced ALF and therefore merits further study. In addition to pyroptosis, necrapoptosis, also known as aponecrosis or apoptotic necrosis, is an important proinflammatory cell death pattern that shared common features and pathways with both apoptosis and necrosis [33, 34]. Also, this cell death pattern was found in liver injury [35, 36]. The necrapoptosis GO term or KEGG pathway was not enriched or mapped in this study. The possible reason might be that this cell demise pattern has not been annotated in databases of GO (http://geneontology.org/) and KEGG (http://www.kegg.jp/), for we cannot retrieve it in either of two databases so far. However, GO term, the adenosine triphosphate (ATP) hydrolysis coupled proton transport, was enriched in this study. ATP has been proved as a key factor to determine the way out of necrapoptosis [34]. This might verify from another aspect in transcriptome level that necrapoptosis is an important cell death pattern involved in the progression of ALF.

Autophagy is a lysosomal pathway tasked with the process of self-degradation of cellular components by the sequestration of these components in double-membrane autophagosomes [37]. It has been widely reported that autophagy plays an important role in cancer and other chronic diseases of the organs [38–42]. Autophagy is an important current research topic in models of liver disease [43–45]. However, currently there are limited data on the role of autophagy in the progression of ALF [46, 47]. In this study, GO terms such as autophagy, regulation of autophagy, and phagocytic-vehicle were all enriched. Two KEGG pathways of lysosomal and phagosome regulation were mapped. These results provide another potential avenue of transcriptome-level research on the influence of autophagy on the progression of ALF.

The role of fibrosis in the progression of chronic liver disease has been widely studied. Although fibrosis is observed in ALF [48], an understanding of the underlying mechanisms remains limited. GO terms related to the collagen-related component of liver fibrosis, such as collagen catabolic processes and collagen binding, were also enriched in this study. Semaphorin families are regulators of the progression of fibrosis in chronic liver diseases [49, 50]. However, the role of semaphorin families in ALF remains unknown. Semaphorin receptor activity GO terms were also found in this study, which constitutes another interesting avenue for research.

In conclusion, this study identified dramatic changes in the PBMC transcriptome predominantly related to immune responses in ALF. Enriched GO terms related to coagulation dysfunction, hepatic encephalopathy, and mapped KEGG pathways of diseases characterised by liver injury demonstrated that the PBMC transcriptome reflects the features of ALF. The enrichment of GO terms related to cell death and fibrosis indicates that PBMC transcriptome analysis is a useful method to elucidate potential key gene sets involved in ALF progression. Thus, a better understanding of the gene sets identified in this study may contribute to ALF prevention or treatment.

## Figures and Tables

**Table 1:** Biochemical parameters in a porcine model of ALF.

Parameters	Baseline	Failure	Dying
International normalization ratio	0.9 ± 0.05	2.7 ± 0.2^*∗∗*^	4.8 ± 0.8^*∗∗*^
Ammonia (*μ*g/dl)	22.3 ± 3.1	76.5 ± 8.7^*∗∗*^	225.5 ± 47.4^*∗∗*^
Alanine aminotransferase (U/L)	56.3 ± 8.0	311.5 ± 65.0^*∗*^	230.3 ± 46.5^*∗*^
Aspartate aminotransferase (U/L)	36.0 ± 3.3	5023.8 ± 1034.6^*∗*^	1788.5 ± 263.6^*∗∗*^
Alkaline phosphatase (U/L)	72.8 ± 16.9	232.8 ± 53.4^*∗*^	564.0 ± 82.6^*∗∗*^
*γ*-Glutamyl transpeptidase (U/L)	64.5 ± 9.6	77.0 ± 5.1	96.3 ± 5.0^*∗*^
Total bilirubin (*μ*mol/L)	2.3 ± 0.3	40.8 ± 5.7^*∗∗*^	70.8 ± 7.6^*∗∗*^
Creatinine (mmol/L)	58.0 ± 2.1	59.3 ± 6.4	49.5 ± 3.3

Data are means ± SEM. ^*∗*^*p* < 0.05, ^*∗∗*^*p* < 0.01 versus baseline.

**Table 2:** Qualitative analysis of PBMC RNA-Seq data in a porcine model of ALF.

Sample name	Raw reads	*Q*20 value	Clean reads	Mapped reads	Genic reads	Percentage of genic reads	Expressed gene number
Baseline-1	80,876,352	94.80%	77,508,054	63,736,589	51,530,237	80.80%	15,249
Baseline-2	122,478,648	95.40%	117,994,584	64,288,782	47,627,719	74.10%	14,990
Baseline-3	97,633,918	95.10%	93,252,338	76,290,007	48,400,655	63.40%	15,470
Baseline-4	91,203,498	95.30%	87,519,976	70,759,871	54,612,207	77.20%	15,642
Dying-1	106,182,528	95.20%	101,728,266	83,553,821	57,816,273	69.20%	15,563
Dying-2	93,997,436	95.20%	90,503,598	74,471,193	60,365,900	81.10%	15,101
Dying-3	118,901,914	94.90%	91,408,790	73,952,809	57,686,330	78.00%	15,712
Dying-4	84,848,674	95.30%	81,623,206	67,004,468	55,869,637	83.40%	15,343
Failure-1	80,262,120	95.20%	76,885,638	63,014,127	49,808,658	79.00%	15,297
Failure-2	100,438,952	95.30%	100,291,397	82,963,827	68,021,072	82.00%	15,812
Failure-3	86,394,661	94.90%	66,295,638	54,080,154	38,129,055	70.50%	15,228
Failure-4	99,697,480	94.80%	95,927,678	79,312,204	60,637,999	76.50%	15,404

**Table 3:** KEGG pathways involved in the progression of ALF.

Name	Failure versus baseline			Dying versus baseline		
	Mapped genes	Fold enrichment	FDR adjusted *p*	Mapped genes	Fold enrichment	FDR adjusted *p*
Cytokine-cytokine receptor interaction	25	3.0	4.3*E* − 04	38	2.5	3.0*E* − 05
Hematopoietic cell lineage	13	4.4	3.8*E* − 03	15	2.7	2.1*E* − 02
Lysosome	14	3.2	2.1*E* − 02	34	4.2	2.9*E* − 10
Rheumatoid arthritis	12	3.7	2.3*E* − 02	21	3.5	7.0*E* − 05
Malaria	9	4.6	2.6*E* − 02	14	3.9	1.4*E* − 03
Phagosome	15	2.7	3.9*E* − 02	26	2.5	1.1*E* − 03
Pertussis	10	3.7	4.4*E* − 02	20	3.9	4.0*E* − 05
NF-kappa B signaling pathway				19	3.1	1.3*E* − 03
Transcriptional misregulation in cancer				26	2.4	1.6*E* − 03
Chagas disease (American trypanosomiasis)				20	2.7	3.3*E* − 03
Leishmaniasis				14	3.3	6.6*E* − 03
TNF signaling pathway				19	2.5	8.5*E* − 03
*Salmonella* infection				16	2.8	8.8*E* − 03
Complement and coagulation cascades				14	2.8	2.1*E* − 02
Mineral absorption				10	3.4	3.1*E* − 02
Osteoclast differentiation				20	2.2	3.2*E* − 02
Legionellosis				12	2.8	4.4*E* − 02
Pentose phosphate pathway				7	4.6	4.6*E* − 02
Histidine metabolism				7	4.6	4.6*E* − 02

## Supplementary Materials

Supplementary Materials Supplementary Table 1: list of differentially expressed genes identified in failure group; Supplementary Table 2: list of differentially expressed genes identified in dying group; Supplementary Table 3: product sizes and the primers used for qRT-PCR; Supplementary Table 4: gene fold change tested by qRT-PCR and RNA-Seq.
