# Supplementary material for: Transcriptome Analysis of Porcine PBMCs Reveals the Immune Cascade Response and Gene Ontology Terms Related to Cell Death and Fibrosis in the Progression of Liver Failure
Source: Can J Gastroenterol Hepatol. 2018 Apr 12;2018:2101906. doi: 10.1155/2018/2101906 (PMC5925156; doi:10.1155/2018/2101906)
Supplement: Supplementary Materials — Supplementary Table 1: list of differentially expressed genes identified in failure group; Supplementary Table 2: list of differentially expressed genes identified in dying group; Supplementary Table 3: product sizes and the primers used for qRT-PCR; Supplementary Table 4: gene fold change tested by qRT-PCR and RNA-Seq. [file 2101906.f1.pdf]

Supplementary Table 1. List of differentially expressed genes identified in failure group

Gene XLOC Number	Gene Symbol/ID	Adjust p
XLOC_000001	DLL1	0.010072
XLOC_000042	ENSSSCG00000024378	0.002372
XLOC_000054	CNKSR3	0.027994
XLOC_000096	-	0.045290
XLOC_000146	SAMD3	0.002372
XLOC_000192	FAM135A	0.002372
XLOC_000211	SLC35A1	0.035659
XLOC_000244	ENSSSCG00000004369	0.002372
XLOC_000330	RAB27B	0.002372
XLOC_000357	RORA	0.002372
XLOC_000359	ANXA2	0.039463
XLOC_000431	C15orf48,ENSSSCG00000018328	0.031419
XLOC_000519	SCG5	0.007395
XLOC_000563	ENSSSCG00000004870	0.002372
XLOC_000579	ENSSSCG00000021694,SERPINB11	0.027058
XLOC_000581	-	0.002372
XLOC_000690	LGALS3	0.011350
XLOC_000694	PELI2	0.020320
XLOC_000716	PKC	0.027058
XLOC_000784	ADFP,ENSSSCG00000005172	0.002372
XLOC_000788	PSIP1	0.008746
XLOC_000845	ANXA1	0.045290
XLOC_000858	PSAT1	0.027058
XLOC_000909	ANP32B	0.010072
XLOC_000917	NOR-1	0.034852
XLOC_000994	TLR4	0.041076
XLOC_001067	STXBP1	0.002372
XLOC_001097	TOR1B	0.029810
XLOC_001130	FCN2	0.018305
XLOC_001231	SYTL3	0.002372
XLOC_001246	ENSSSCG00000004072	0.045290
XLOC_001252	SYNE1	0.002372
XLOC_001263	LRP11	0.033125
XLOC_001278	RAB32	0.045290
XLOC_001282	-	0.002372
XLOC_001283	ENSSSCG00000026681	0.012580
XLOC_001285	STX11	0.002372
XLOC_001303	CCDC28A	0.036488
XLOC_001361	SMPDL3A	0.008746

XLOC_001374	NUS1	0.044005
XLOC_001412	CGA	0.007395
XLOC_001424	RRAGD	0.002372
XLOC_001429	-	0.016103
XLOC_001509	FAM26F	0.002372
XLOC_001541	PSTPIP2	0.035659
XLOC_001584	ENSSSCG00000004560	0.015000
XLOC_001585	DAPK2	0.002372
XLOC_001617	AQP9	0.019332
XLOC_001651	SEMA6D	0.004177
XLOC_001654	SLC28A2	0.002372
XLOC_001682	SP23	0.022312
XLOC_001688	ENSSSCG00000020666	0.022312
XLOC_001723	THBS1	0.002372
XLOC_001725	SPRED1	0.005845
XLOC_001796	SERPINB8	0.015000
XLOC_001797	SERPINB10	0.002372
XLOC_001802	TNFRSF11A	0.017207
XLOC_001823	RAB11A	0.029810
XLOC_001845	ENSSSCG00000004963	0.007395
XLOC_001859	PNN	0.002372
XLOC_001892	GNG2	0.038749
XLOC_001902	BMP4	0.036488
XLOC_001933	ISG20	0.002372
XLOC_001974	ACER2	0.007395
XLOC_002012	Metazoa_SRP	0.004177
XLOC_002028	MAMDC2	0.025115
XLOC_002063	Rse_MRP	0.002372
XLOC_002099	CORO2A,TBC1D2	0.020320
XLOC_002100	ENSSSCG00000005377,ENSSSCG00000027558	0.002372
XLOC_002140	TRX1	0.002372
XLOC_002183	ENSSSCG00000028117	0.019332
XLOC_002184	MEGF9	0.002372
XLOC_002258	FAM129B	0.038749
XLOC_002320	FCN1	0.021314
XLOC_002611	-	0.034852
XLOC_002663	-	0.030613
XLOC_002685	-	0.037305
XLOC_002776	-	0.005845
XLOC_002863	-	0.004177
XLOC_003355	-	0.041848
XLOC_003356	-	0.033995
XLOC_003473	-	0.002372

XLOC_003946	CNIH4	0.015000
XLOC_003991	DENND1B	0.004177
XLOC_004019	FBP1	0.002372
XLOC_004051	AQP3	0.045889
XLOC_004068	C9orf72	0.002372
XLOC_004096	-	0.032252
XLOC_004105	-	0.032252
XLOC_004196	KLF6	0.025115
XLOC_004224	RGS2	0.039463
XLOC_004225	RGS1	0.002372
XLOC_004228	RGS18	0.004177
XLOC_004443	FRMD4A	0.040229
XLOC_004477	NRP1	0.002372
XLOC_004492	GATA3	0.002372
XLOC_004736	-	0.021314
XLOC_004941	-	0.042609
XLOC_004983	-	0.019332
XLOC_005126	-	0.021314
XLOC_005174	SLC46A3	0.002372
XLOC_005184	ALOX5AP	0.010072
XLOC_005201	ENSSSCG00000021829	0.030613
XLOC_005214	LHFP	0.044653
XLOC_005456	-	0.008746
XLOC_005472	CDADC1	0.022312
XLOC_005499	TNFSF11	0.015000
XLOC_005530	-	0.028943
XLOC_005537	-	0.011350
XLOC_005550	SPRY2	0.012580
XLOC_005568	ENSSSCG00000028678	0.007395
XLOC_005590	ENSSSCG00000009518	0.043224
XLOC_005623	RAB20	0.012580
XLOC_005629	LAMP1	0.033995
XLOC_005637	GAS6	0.012580
XLOC_006065	FN3K,FN3KRP	0.045290
XLOC_006094	TIMP-2	0.004177
XLOC_006097	-	0.004177
XLOC_006099	SCAR16	0.007395
XLOC_006206	PLEKHM1	0.010072
XLOC_006227	GRN	0.004177
XLOC_006237	PYY	0.018305
XLOC_006249	RND2	0.025115
XLOC_006251	TMEM106A	0.002372
XLOC_006289	TOP2A	0.008746
XLOC_006378	ABCC3	0.007395

XLOC_006388	TOM1L1	0.015000
XLOC_006465	CCL23	0.002372
XLOC_006466	CCL16	0.002372
XLOC_006467	CCL5	0.002372
XLOC_006472	ENSSSCG00000024115	0.030613
XLOC_006484	CCL8	0.002372
XLOC_006485	CCL2	0.002372
XLOC_006506	ENSSSCG00000017754	0.002372
XLOC_006581	SPNS3	0.008746
XLOC_006596	CXCL16	0.036488
XLOC_006635	CHD3	0.002372
XLOC_006728	SEC14L1	0.048079
XLOC_006734	SPHK1	0.004177
XLOC_006793	AXIN2	0.019332
XLOC_006844	ITGA2B	0.002372
XLOC_006856	VAT1	0.044005
XLOC_006866	RAMP2	0.022312
XLOC_006965	TMEM92	0.020320
XLOC_006993	SCPEP1	0.004177
XLOC_007043	SNORA11	0.043224
XLOC_007074	ADAP2	0.002372
XLOC_007086	NOS2	0.004177
XLOC_007117	SLC6A4	0.002372
XLOC_007130	FAM101B	0.016103
XLOC_007169	ENSSSCG00000022405	0.015000
XLOC_007180	XAF1	0.047338
XLOC_007186	WSCD1	0.002372
XLOC_007195	GPIBA	0.004177
XLOC_007220	ENSSSCG00000025782	0.012580
XLOC_007486	-	0.017207
XLOC_007527	-	0.044653
XLOC_007658	-	0.002372
XLOC_007930	EIF1B	0.002372
XLOC_007972	CCR2	0.004177
XLOC_007973	CCR5	0.010072
XLOC_007974	CCRL2	0.022312
XLOC_007978	PTH1R	0.004177
XLOC_008183	-	0.002372
XLOC_008184	DJC13	0.036488
XLOC_008198	CEP63	0.012580
XLOC_008234	ENSSSCG00000011676	0.030613
XLOC_008261	P2RY1	0.004177
XLOC_008279	ENSSSCG00000027556	0.021314
XLOC_008283	MFSD1	0.007395

XLOC_008288	-	0.018305
XLOC_008299	-	0.016103
XLOC_008306	-	0.002372
XLOC_008402	IL1RAP	0.008746
XLOC_008410	HES1	0.017207
XLOC_008438	OSBPL11	0.005845
XLOC_008486	UBE2B	0.008746
XLOC_008549	ENSSSCG00000025392	0.002372
XLOC_008556	GBE1	0.002372
XLOC_008557	-	0.004177
XLOC_008562	ENSSSCG00000012006	0.020320
XLOC_008619	BACE2	0.013835
XLOC_008658	-	0.002372
XLOC_008665	ANKRD28	0.033995
XLOC_008698	-	0.026101
XLOC_008737	CX3CR1	0.002372
XLOC_008772	CCR1	0.002372
XLOC_008773	CCR2	0.002372
XLOC_008774	LTF	0.002372
XLOC_008809	ENSSSCG00000011361	0.002372
XLOC_008812	SLC25A20	0.002372
XLOC_008826	UBA7	0.035659
XLOC_008840	MAPKAPK3	0.004177
XLOC_008872	TKT	0.002372
XLOC_008990	TMEM40	0.007395
XLOC_008992	PLXND1	0.037305
XLOC_009004	FBLN2	0.022312
XLOC_009030	ACPP	0.008746
XLOC_009103	SIAH2	0.015000
XLOC_009109	P2RY13	0.002372
XLOC_009150	BCHE	0.002372
XLOC_009185	-	0.011350
XLOC_009197	B3GNT5	0.005845
XLOC_009239	MB21D2	0.044005
XLOC_009273	MYLK	0.010072
XLOC_009275	-	0.002372
XLOC_009289	ENSSSCG00000027867	0.002372
XLOC_009322	ATP6V1A,GRAMD1C	0.037305
XLOC_009342	TRAT1	0.030613
XLOC_009401	CRYBG3,ENSSSCG00000022895	0.002372
XLOC_009405	ENSSSCG00000011993	0.002372
XLOC_009414	ENSSSCG00000029384	0.002372
XLOC_009468	ENSSSCG00000024614	0.040229
XLOC_009489	-	0.048079

XLOC_009518	TRPM2	0.045889
XLOC_010021	-	0.020320
XLOC_010181	-	0.005845
XLOC_010667	-	0.002372
XLOC_010848	-	0.005845
XLOC_011044	ENSSSCG00000009627	0.024202
XLOC_011174	CCDC63	0.007395
XLOC_011178	SH2B3	0.027058
XLOC_011204	PLBD2	0.029810
XLOC_011210	OAS2	0.002372
XLOC_011222	U4	0.002372
XLOC_011230	MLEC	0.037305
XLOC_011249	CMLKR1	0.002372
XLOC_011365	GP1BB	0.016103
XLOC_011373	ENSSSCG00000026176	0.027994
XLOC_011406	TTC13	0.013835
XLOC_011536	-	0.048079
XLOC_011557	MINPP1	0.021314
XLOC_011567	IFIT2	0.002372
XLOC_011568	IFIT3	0.002372
XLOC_011569	IFIT1	0.004177
XLOC_011570	IFIT5	0.011350
XLOC_011579	HHEX	0.023280
XLOC_011589	SLC35G1	0.002372
XLOC_011599	ENSSSCG00000010498,ENTPD1	0.004177
XLOC_011819	ENTPD4	0.019332
XLOC_011978	ENSSSCG00000009926	0.002372
XLOC_012015	OSM	0.002372
XLOC_012029	PATZ1,PIK3IP1	0.041076
XLOC_012128	RHOU	0.021314
XLOC_012142	ANK3	0.046618
XLOC_012180	PRF1	0.002372
XLOC_012221	TMEM254	0.004177
XLOC_012271	ALOX5	0.002372
XLOC_012278	PRKG1	0.020320
XLOC_012285	ANKRD22	0.002372
XLOC_012288	LIPA	0.037305
XLOC_012301	ENSSSCG00000010476	0.033125
XLOC_012337	SLC25A28	0.008746
XLOC_012442	CHST15	0.002372
XLOC_013061	-	0.017207
XLOC_013248	CXCR4	0.002372
XLOC_013276	MARCO	0.002372
XLOC_013283	BIN1	0.002372

XLOC_013297	PTPN4	0.049392
XLOC_013306	KBTBD11	0.004177
XLOC_013333	CASP3	0.044653
XLOC_013394	G3PD	0.002372
XLOC_013402	ENSSSCG00000015880	0.032252
XLOC_013497	-	0.005845
XLOC_013517	ENSSSCG00000027157	0.002372
XLOC_013518	ENSSSCG00000016040	0.002372
XLOC_013533	ENSSSCG00000016063,ENSSSCG00000026618	0.007395
XLOC_013547	PLCL1	0.002372
XLOC_013583	FAM117B	0.010072
XLOC_013641	NRAMP1	0.002372
XLOC_013812	-	0.020320
XLOC_013824	HNMT	0.002372
XLOC_013826	-	0.049392
XLOC_013900	FACL2	0.048740
XLOC_013910	FAM149A	0.008746
XLOC_013975	NR4A2	0.027058
XLOC_014072	TFPI	0.019332
XLOC_014077	ENSSSCG00000026081	0.002372
XLOC_014081	ENSSSCG00000027221	0.002372
XLOC_014089	STAT1	0.040229
XLOC_014090	STAT4	0.033125
XLOC_014123	ENSSSCG00000016106	0.030613
XLOC_014160	IDH1	0.002372
XLOC_014170	FN1	0.002372
XLOC_014177	ENSSSCG00000027669	0.005845
XLOC_014179	ENSSSCG00000016184	0.049392
XLOC_014237	DNER	0.049392
XLOC_014263	-	0.029810
XLOC_014271	SCAR6	0.004177
XLOC_014941	-	0.017207
XLOC_015077	EMB,SNORD28	0.034852
XLOC_015081	ITGA2	0.002372
XLOC_015088	GZMA	0.008746
XLOC_015103	-	0.007395
XLOC_015112	ENSSSCG00000016938	0.038059
XLOC_015127	LY64	0.002372
XLOC_015150	-	0.049392
XLOC_015198	RNF145	0.029810
XLOC_015204	ADAM19	0.010072
XLOC_015249	CH242-128B1.2	0.012580
XLOC_015324	DAB2	0.039463

XLOC_015339	-	0.004177
XLOC_016059	AP3M2	0.002372
XLOC_016098	PC2	0.019332
XLOC_016127	CD93	0.002372
XLOC_016139	ENSSSCG00000023107	0.005845
XLOC_016145	SIGLEC1	0.007395
XLOC_016302	SLPI	0.002372
XLOC_016303	ENSSSCG00000025317,PTI	0.036488
XLOC_016314	MMP9	0.002372
XLOC_016343	C/EBP,CEBPB	0.017207
XLOC_016414	MSR1	0.002372
XLOC_016418	ENSSSCG00000006989	0.011350
XLOC_016424	ASAH1	0.030613
XLOC_016434	IDO1	0.002372
XLOC_016459	PRP	0.002372
XLOC_016465	GPCPD1	0.022312
XLOC_016492	SNORD17	0.002372
XLOC_016521	SMOX	0.002372
XLOC_016666	ENSSSCG00000022258	0.002372
XLOC_016700	SULF2	0.002372
XLOC_016703	ENSSSCG00000022915	0.045889
XLOC_016706	DRK1	0.002372
XLOC_016709	B4GALT5	0.010072
XLOC_016820	-	0.011350
XLOC_017187	AOC1	0.020320
XLOC_017188	GIMAP1	0.047338
XLOC_017233	CREB3L2	0.021314
XLOC_017304	TSPAN12	0.002372
XLOC_017313	TFEC	0.002372
XLOC_017393	ENSSSCG00000016732,SNORA5	0.002372
XLOC_017416	PSMA2	0.011350
XLOC_017459	GALNTL5	0.011350
XLOC_017499	ENSSSCG00000029386	0.038059
XLOC_017516	TBXAS1	0.049392
XLOC_017596	GPR37	0.002372
XLOC_017680	ENSSSCG00000023695	0.020320
XLOC_017682	SNX10	0.022312
XLOC_018104	-	0.011350
XLOC_018122	-	0.025115
XLOC_018272	CPT1A	0.002372
XLOC_018278	ENSSSCG00000028501	0.008746
XLOC_018344	BATF2	0.002372
XLOC_018432	MPEG1	0.002372
XLOC_018494	C1QTNF4	0.030613

XLOC_018565	LGR4	0.002372
XLOC_018566	CCDC34	0.008746
XLOC_018585	ENSSSCG00000021688	0.022312
XLOC_018606	PIK3C2A	0.002372
XLOC_018634	LYVE1	0.019332
XLOC_018702	ENSSSCG00000027848	0.038749
XLOC_018727	ENSSSCG00000024211	0.018305
XLOC_018866	RETN	0.002372
XLOC_018867	-	0.004177
XLOC_018880	ADGRE1,ENSSSCG00000022228	0.002372
XLOC_018928	MFSD12	0.040229
XLOC_018954	ARID3A	0.013835
XLOC_018999	ADAMTS2	0.013835
XLOC_019017	HK3	0.002372
XLOC_019051	LHFPL2	0.004177
XLOC_019073	VCAN	0.004177
XLOC_019122	CAMK4,STARD4	0.041848
XLOC_019155	SNX24	0.027058
XLOC_019235	CTN1	0.004177
XLOC_019238	SNORA74	0.015000
XLOC_019297	ABLIM3	0.002372
XLOC_019369	ATGL	0.025115
XLOC_019400	CPT1A	0.034852
XLOC_019446	CTSW	0.002372
XLOC_019538	SLC15A3	0.048740
XLOC_019570	FAM111A	0.033125
XLOC_019571	FAM111B	0.002372
XLOC_019690	CD59	0.002372
XLOC_019737	NUCB2	0.025115
XLOC_019753	-	0.017207
XLOC_019765	ADM	0.002372
XLOC_019825	B3GNT3	0.030613
XLOC_019847	ENSSSCG00000013842,ENSSSCG00000027903	0.002372
XLOC_019970	RAB3D	0.002372
XLOC_020010	-	0.005845
XLOC_020038	UHRF1	0.044005
XLOC_020072	GADD45B	0.004177
XLOC_020157	ENC1	0.043224
XLOC_020198	-	0.019332
XLOC_020208	-	0.026101
XLOC_020237	LIX1	0.002372
XLOC_020370	CD14	0.002372
XLOC_020383	ARAP3	0.002372

XLOC_020417	-	0.004177
XLOC_021078	-	0.028943
XLOC_021079	-	0.010072
XLOC_021428	TTYH3	0.034852
XLOC_021497	ENSSSCG00000023912	0.002372
XLOC_021550	-	0.002372
XLOC_021554	TRIM72	0.002372
XLOC_021574	-	0.002372
XLOC_021586	SPNS1	0.016103
XLOC_021667	ROGDI	0.002372
XLOC_021693	-	0.049392
XLOC_021737	SLC9A3R2	0.002372
XLOC_021751	ATP6V0C	0.029810
XLOC_021783	ENSSSCG00000022493	0.002372
XLOC_021810	CCDC138	0.016103
XLOC_021814	NCK2	0.049392
XLOC_021823	IL1R2	0.002372
XLOC_021824	-	0.002372
XLOC_021868	CD8B	0.043224
XLOC_021874	-	0.010072
XLOC_021882	NKL	0.002372
XLOC_021958	-	0.020320
XLOC_021966	RAB1B	0.027058
XLOC_022009	ENSSSCG00000008409,RTN4	0.024202
XLOC_022195	CMPK2	0.004177
XLOC_022222	PDGFA	0.023280
XLOC_022337	TPST1	0.019332
XLOC_022353	COX6A2	0.024202
XLOC_022354	-	0.030613
XLOC_022357	ENSSSCG00000007758	0.026101
XLOC_022515	IL9R	0.015000
XLOC_022592	IL1A	0.030613
XLOC_022634	FHL2	0.010072
XLOC_022644	IL1RL1	0.002372
XLOC_022647	IL1R2	0.002372
XLOC_022689	CD8A	0.004177
XLOC_022721	RPL7A	0.044653
XLOC_022725	HK2	0.002372
XLOC_022761	GK	0.002372
XLOC_022773	ANXA4	0.004177
XLOC_022795	ENSSSCG00000030005	0.002372
XLOC_022893	ENSSSCG00000008491	0.008746
XLOC_022920	YPEL5	0.011350
XLOC_022955	SLC35F6	0.023280

XLOC_023010	-	0.016103
XLOC_023012	IRG6	0.032252
XLOC_023481	-	0.018305
XLOC_023674	-	0.002372
XLOC_024100	ENSSSCG00000006954	0.007395
XLOC_024107	SLC45A4	0.007395
XLOC_024191	ANGPT1	0.045889
XLOC_024205	NCALD	0.004177
XLOC_024271	CA1	0.002372
XLOC_024281	ZBTB10	0.036488
XLOC_024313	SNORD87	0.002372
XLOC_024327	GGH	0.011350
XLOC_024369	SELP	0.002372
XLOC_024370	F5	0.007395
XLOC_024381	CD3Z	0.002372
XLOC_024415	SLAMF7	0.016103
XLOC_024531	S100A9	0.002372
XLOC_024563	FAM63A	0.046618
XLOC_024593	PRKAB2	0.021314
XLOC_024605	ZNF697	0.021314
XLOC_024626	TSPAN2	0.007395
XLOC_024639	SLC16A1	0.036488
XLOC_024669	SORT1	0.005845
XLOC_024780	GSDMD	0.031419
XLOC_024793	ENSSSCG00000005941	0.037305
XLOC_024810	SQLE	0.044653
XLOC_024842	AARD	0.002372
XLOC_024876	KLF10	0.046618
XLOC_024898	PGCP	0.005845
XLOC_024900	SDC2	0.002372
XLOC_025058	CD32	0.004177
XLOC_025141	LM	0.005845
XLOC_025193	S100A12	0.002372
XLOC_025194	S100A8	0.002372
XLOC_025214	TNFAIP8L2	0.030613
XLOC_025218	PRUNE	0.011350
XLOC_025223	ECM1	0.002372
XLOC_025263	ENSSSCG00000030938,PHGDH	0.004177
XLOC_025284	MAB21L3,SLC22A15	0.010072
XLOC_025619	-	0.002372
XLOC_025832	-	0.023280
XLOC_026020	-	0.012580
XLOC_026339	-	0.007395
XLOC_026397	ENSSSCG00000029668	0.002372

XLOC_026459	ACVRL1	0.007395
XLOC_026460	ACVR1B	0.011350
XLOC_026472	EIF4B	0.029810
XLOC_026589	SLC16A7	0.002372
XLOC_026616	IFN-GAMMA	0.010072
XLOC_026710	-	0.017207
XLOC_026717	LRP6	0.002372
XLOC_026726	ENSSSCG00000000639	0.038749
XLOC_026727	ENSSSCG00000000640	0.002372
XLOC_026728	KLRK1	0.002372
XLOC_026739	CLECL1	0.004177
XLOC_026741	KLRB1	0.004177
XLOC_026746	KLRG1	0.002372
XLOC_026750	SLC2A3	0.013835
XLOC_026753	ENSSSCG00000026009	0.002372
XLOC_026767	TNFRSF1A	0.042609
XLOC_026768	VWF	0.002372
XLOC_026803	USP18	0.012580
XLOC_026893	LTA4H	0.041848
XLOC_026903	PLXNC1	0.040229
XLOC_026921	ATP2B1	0.032252
XLOC_026945	DYRK2	0.011350
XLOC_027110	PF4,PPBP	0.012580
XLOC_027144	NFE2	0.033995
XLOC_027145	ENSSSCG00000000291	0.002372
XLOC_027181	MMP19	0.005845
XLOC_027256	-	0.008746
XLOC_027268	LYZ	0.002372
XLOC_027330	PDE3A	0.041848
XLOC_027350	PLBD1	0.002372
XLOC_027353	HEBP1	0.047338
XLOC_027356	BCL2L14	0.004177
XLOC_027367	CLEC1B	0.002372
XLOC_027378	ENSSSCG00000025680	0.002372
XLOC_027410	KC6	0.047338
XLOC_027427	ENSSSCG00000000744,FKBP4	0.031419
XLOC_027459	TWF1	0.010072
XLOC_027505	DRAM1	0.002372
XLOC_027506	CHPT1	0.033125
XLOC_027554	ENSSSCG00000000921	0.002372
XLOC_027606	-	0.027994
XLOC_027691	-	0.029810
XLOC_027930	-	0.002372
XLOC_027941	-	0.033995

XLOC_027950	-	0.002372
XLOC_027954	-	0.040229
XLOC_028073	-	0.048079
XLOC_028322	GPR56	0.002372
XLOC_028323	ADGRG3	0.010072
XLOC_028380	ENSSSCG00000026318	0.002372
XLOC_028482	RPS16	0.038749
XLOC_028575	C5AR1	0.002372
XLOC_028576	C5AR2,DHX34	0.035659
XLOC_028645	ENSSSCG00000003226	0.018305
XLOC_028680	-	0.036488
XLOC_028820	PGD	0.002372
XLOC_028836	MFN2	0.012580
XLOC_028839	-	0.039463
XLOC_028868	PADI4	0.002372
XLOC_028870	ENSSSCG00000022946	0.043224
XLOC_028885	CDA	0.002372
XLOC_028924	ENSSSCG00000024625,STMN1	0.016103
XLOC_028954	SMPDL3B	0.002372
XLOC_028972	SDC3	0.002372
XLOC_029015	UTP11L	0.018305
XLOC_029022	ENSSSCG00000027920	0.024202
XLOC_029199	ZFYVE9	0.024202
XLOC_029254	-	0.036488
XLOC_029322	KIAA0513	0.002372
XLOC_029400	CCDC102A	0.011350
XLOC_029426	ATP6V0D1	0.027058
XLOC_029472	C/EBP	0.002372
XLOC_029484	FFAR2	0.002372
XLOC_029570	AXL	0.002372
XLOC_029610	PVR	0.048740
XLOC_029619	PTGIR	0.002372
XLOC_029701	NKG7	0.007395
XLOC_029708	ENSSSCG00000024518,SIGLEC5	0.005845
XLOC_029722	NLRP12	0.002372
XLOC_029727	TARM1	0.002372
XLOC_029898	PADI2	0.002372
XLOC_029900	ARHGEF10L	0.004177
XLOC_029922	ECE1	0.002372
XLOC_029936	PITHD1	0.004177
XLOC_029943	RUNX3	0.011350
XLOC_029944	-	0.002372
XLOC_030014	AZIN2	0.023280

XLOC_030054	MYCL	0.002372
XLOC_030079	ENSSSCG00000003686	0.004177
XLOC_030080	EPB41L3	0.004177
XLOC_030107	NPC1	0.024202
XLOC_030132	ENSSSCG00000029762	0.024202
XLOC_030143	ENSSSCG00000025209	0.002372
XLOC_030151	ADGRL2	0.002372
XLOC_030153	IFI44	0.007395
XLOC_030233	ZYG11A	0.002372
XLOC_030751	-	0.002372
XLOC_031046	-	0.002372
XLOC_031297	-	0.015000
XLOC_031298	-	0.007395
XLOC_031468	EDN1	0.013835
XLOC_031480	GMPR	0.029810
XLOC_031493	SOX4	0.030613
XLOC_031522	ZNF322	0.007395
XLOC_031574	GABBR1	0.018305
XLOC_031575	UBD	0.007395
XLOC_031680	ANKS1A	0.026101
XLOC_031710	LRFN2	0.032252
XLOC_031726	-	0.026101
XLOC_031781	CRISP3	0.015000
XLOC_031804	FAH	0.039463
XLOC_031808	MESDC2	0.043224
XLOC_031889	HEXA	0.016103
XLOC_031892	PKM	0.020320
XLOC_031916	EGLN3	0.002372
XLOC_031933	GZMH	0.013835
XLOC_031960	CEBPE	0.010072
XLOC_032093	PSEN1	0.011350
XLOC_032113	FOS	0.002372
XLOC_032271	SERPINB1	0.002372
XLOC_032314	ENSSSCG00000020858	0.004177
XLOC_032321	MBOAT1	0.002372
XLOC_032460	AIF1	0.002372
XLOC_032464	TNFA	0.002372
XLOC_032566	ETV7	0.008746
XLOC_032593	TREML1	0.004177
XLOC_032647	TNFRSF21	0.042609
XLOC_032732	C7H15orf38	0.005845
XLOC_032744	SCAPER	0.046618
XLOC_032809	GZMB,GZMH	0.002372
XLOC_032851	TRDC	0.002372

XLOC_032857	TRADV8	0.002372
XLOC_032943	SLCO3A1	0.002372
XLOC_032958	ATP6V1D	0.034852
XLOC_032985	ENSSSCG00000022178	0.002372
XLOC_033008	-	0.021314
XLOC_033030	-	0.045290
XLOC_033033	GALC	0.048740
XLOC_033059	ENSSSCG00000002452	0.002372
XLOC_033087	BCL11B	0.041848
XLOC_033093	WARS	0.024202
XLOC_033094	CDC42BPB	0.013835
XLOC_033257	-	0.023280
XLOC_034070	JAKMIP1	0.010072
XLOC_034099	ENSSSCG00000028448	0.010072
XLOC_034114	RBPJ	0.012580
XLOC_034124	ENSSSCG00000008769	0.015000
XLOC_034206	TMEM144	0.004177
XLOC_034226	HOPX	0.004177
XLOC_034259	UTP3	0.048740
XLOC_034261	MOB1B	0.039463
XLOC_034263	SLC4A4	0.002372
XLOC_034293	U1	0.016103
XLOC_034376	TRPC3	0.002372
XLOC_034380	ANXA5	0.010072
XLOC_034391	PDE5A	0.002372
XLOC_034489	HPSE	0.002372
XLOC_034491	ENSSSCG00000009239	0.002372
XLOC_034565	LYAR	0.019332
XLOC_034584	ENSSSCG00000023604	0.004177
XLOC_034585	-	0.002372
XLOC_034611	TLR10	0.015000
XLOC_034612	TLR1,TLR6	0.042609
XLOC_034624	RBM47	0.007395
XLOC_034633	GNPDA2	0.002372
XLOC_034707	-	0.048740
XLOC_034748	-	0.002372
XLOC_034758	ALB	0.002372
XLOC_034770	AA	0.002372
XLOC_034776	SCARB2	0.015000
XLOC_034830	GAB1	0.038749
XLOC_034923	SLC39A8	0.048740
XLOC_034953	ENSSSCG00000030548	0.010072
XLOC_034964	ENSSSCG00000009226	0.034852
XLOC_034968	PTPN13	0.002372

XLOC_034972	CDS1	0.002372
XLOC_034973	AGPAT9	0.019332
XLOC_035238	-	0.039463
XLOC_035732	-	0.044005
XLOC_035741	TPP1	0.021314
XLOC_035820	FOLR1	0.002372
XLOC_035863	CAPN5	0.027994
XLOC_035885	-	0.002372
XLOC_035937	ENDOD1	0.049392
XLOC_035970	RAB39A	0.007395
XLOC_035981	ZC3H12C	0.002372
XLOC_036011	CADM1	0.049392
XLOC_036027	CD3E	0.002372
XLOC_036050	OAF	0.005845
XLOC_036082	VWA5A	0.004177
XLOC_036169	RAB7B	0.016103
XLOC_036170	-	0.002372
XLOC_036171	C1orf186	0.002372
XLOC_036179	-	0.013835
XLOC_036189	C4BPA,ENSSSCG00000015663	0.002372
XLOC_036208	GATAD1	0.035659
XLOC_036217	GNG11	0.002372
XLOC_036253	-	0.026101
XLOC_036293	HGF	0.002372
XLOC_036332	ENSSSCG00000027699	0.031419
XLOC_036347	-	0.027994
XLOC_036419	RGL1	0.004177
XLOC_036432	PLA2G4A	0.021314
XLOC_036443	ATF3	0.002372
XLOC_036593	ENSSSCG00000014727	0.030613
XLOC_036594	HBE1	0.010072
XLOC_036607	OR51E1	0.002372
XLOC_036683	PAK1	0.004177
XLOC_036707	SYTL2	0.002372
XLOC_036773	CASP1,ENSSSCG00000014997	0.038059
XLOC_036904	ENSSSCG00000015200	0.005845
XLOC_036948	SOX13	0.002372
XLOC_036957	LRRN2	0.040229
XLOC_036966	SLC45A3	0.002372
XLOC_037017	TFPI2	0.004177
XLOC_037026	PON2	0.027058
XLOC_037040	ETV1	0.002372
XLOC_037051	TSPAN13	0.002372

XLOC_037088	CD36	0.017207
XLOC_037122	ENSSSCG00000023584,ENSSSCG00000026913,ENSSSCG00000029596,TRGV11	0.005845
XLOC_037123	AMPH	0.004177
XLOC_037125	Y_R	0.023280
XLOC_037149	CHI3L1	0.002372
XLOC_037156	FASLG	0.019332
XLOC_037196	XPR1	0.002372
XLOC_037208	RSEL	0.005845
XLOC_037214	NCF2	0.033125
XLOC_037235	FLVCR1	0.015000
XLOC_037259	-	0.040229
XLOC_037307	-	0.002372
XLOC_037604	-	0.020320
XLOC_037889	-	0.002372
XLOC_038083	-	0.043224
XLOC_038183	-	0.020320
XLOC_038283	ALS2	0.027994
XLOC_038373	-	0.027058
XLOC_038567	-	0.036488
XLOC_038672	DAGLB	0.005845
XLOC_038685	ENSSSCG00000021949	0.002372
XLOC_038714	ENSSSCG00000028099	0.002372
XLOC_038767	ANG1,RSE4	0.002372
XLOC_038828	ENSSSCG00000025390	0.047338
XLOC_038902	CPNE2	0.004177
XLOC_039051	ASAP3	0.024202
XLOC_039081	-	0.045889
XLOC_039085	-	0.016103
XLOC_039086	-	0.007395
XLOC_039087	-	0.040229
XLOC_039109	GZMM	0.002372
XLOC_039241	-	0.047338
XLOC_039369	ENSSSCG00000028686	0.041848
XLOC_039706	-	0.027058
XLOC_040136	ENSSSCG00000029802	0.002372
XLOC_040309	ENSSSCG00000022360	0.002372
XLOC_040317	-	0.041076
XLOC_040352	ENSSSCG00000022971	0.031419
XLOC_040495	-	0.004177
XLOC_040497	RSE6	0.002372
XLOC_040569	-	0.002372

XLOC_040662	DAPK2	0.011350
XLOC_040793	SERPINE1	0.002372
XLOC_040957	HMOX1	0.002372
XLOC_040967	-	0.004177
XLOC_040968	UPP1	0.028943
XLOC_040978	SLC2A5	0.008746
XLOC_040979	-	0.002372
XLOC_040987	ENSSSCG00000020872	0.021314
XLOC_041085	-	0.032252
XLOC_041111	PTMA	0.047338
XLOC_041120	-	0.020320
XLOC_041128	PROS1	0.002372
XLOC_041169	-	0.005845
XLOC_041230	-	0.044653
XLOC_041231	-	0.027994
XLOC_041268	-	0.002372
XLOC_041413	-	0.008746
XLOC_041496	CKB	0.002372
XLOC_041501	ENSSSCG00000026336	0.002372
XLOC_041512	SGIP1	0.035659
XLOC_041613	SLC37A3	0.038059
XLOC_041623	-	0.044653
XLOC_041668	HIATL1	0.012580
XLOC_041687	ENSSSCG00000029732	0.002372
XLOC_041737	-	0.002372
XLOC_041875	-	0.039463
XLOC_041922	-	0.029810
XLOC_041962	ENSSSCG00000021402	0.002372
XLOC_042011	WDR73	0.008746
XLOC_042052	ENSSSCG00000021766	0.016103
XLOC_042177	-	0.002372
XLOC_042222	-	0.038749
XLOC_042272	-	0.022312
XLOC_042316	EMR4	0.017207
XLOC_042323	S100B	0.004177
XLOC_042483	APOBR	0.022312
XLOC_042590	CSF2RA	0.002372
XLOC_042606	IGHE	0.045889
XLOC_042621	-	0.011350
XLOC_042713	C3AR1	0.002372
XLOC_042757	SNRPN	0.038749
XLOC_042819	PRSS57	0.027058
XLOC_042822	-	0.023280
XLOC_043048	-	0.002372

XLOC_043100	-	0.004177
XLOC_043210	ENSSSCG00000001071	0.033125
XLOC_043278	ENSSSCG00000025003	0.040229
XLOC_043402	NRP2	0.004177
XLOC_043491	ABHD10	0.011350
XLOC_043495	DSC2	0.032252
XLOC_043601	ENSSSCG00000021096	0.031419
XLOC_043669	-	0.002372
XLOC_043886	STAT1	0.022312
XLOC_044313	BMX	0.027058
XLOC_044370	GK	0.002372
XLOC_044394	CYBB,XK	0.004177
XLOC_044401	ENSSSCG00000012238	0.020320
XLOC_044406	ATP6AP2	0.035659
XLOC_044433	CFP,CH242-69I19.6,ELK1,UXT	0.039463
XLOC_044461	CLCN5	0.005845
XLOC_044476	TSPYL2	0.004177
XLOC_044506	-	0.002372
XLOC_044508	VSIG4	0.002372
XLOC_044509	HEPH	0.031419
XLOC_044517	PJA1	0.007395
XLOC_044547	ENSSSCG00000012411	0.049392
XLOC_044572	ENSSSCG00000012439	0.037305
XLOC_044582	-	0.018305
XLOC_044641	BHLHB9,GPRASP2	0.005845
XLOC_044718	-	0.031419
XLOC_044735	SH2D1A	0.010072
XLOC_044921	SCML2	0.020320
XLOC_044958	CXorf21	0.036488
XLOC_044968	TMEM47	0.002372
XLOC_045005	MAOB	0.002372
XLOC_045097	ARHGEF9	0.015000
XLOC_045103	ENSSSCG00000028241	0.002372
XLOC_045287	LONRF3	0.002372
XLOC_045292	KLHL13	0.005845
XLOC_045393	L1CAM	0.025115
XLOC_045420	-	0.010072

Supplementary Table 2. List of differentially expressed genes identified in dying group

Gene XLOC Number	Gene Symbol/ID	Adjust p
XLOC_000001	DLL1	0.002372
XLOC_000013	ENSSSCG00000004024	0.024202
XLOC_000032	-	0.008746
XLOC_000042	ENSSSCG00000024378	0.002372
XLOC_000062	ENSSSCG00000004082,ENSSSCG00000021273	0.012580
XLOC_000070	ULBP1	0.002372
XLOC_000108	IFNGR1	0.002372
XLOC_000116	PDE7B	0.012580
XLOC_000117	AHI1	0.016103
XLOC_000146	SAMD3	0.002372
XLOC_000151	THEMIS	0.005845
XLOC_000152	-	0.002372
XLOC_000192	FAM135A	0.002372
XLOC_000195	OGFRL1	0.021314
XLOC_000211	SLC35A1	0.002372
XLOC_000262	CDC40	0.011350
XLOC_000273	MARCKS	0.002372
XLOC_000283	IBTK	0.017207
XLOC_000284	FAM46A	0.004177
XLOC_000296	U6	0.002372
XLOC_000305	SETBP1	0.030613
XLOC_000342	OAZ2	0.011350
XLOC_000357	RORA	0.002372
XLOC_000359	ANXA2	0.002372
XLOC_000360	ENSSSCG00000004582	0.046618
XLOC_000362	MYO1E	0.010072
XLOC_000382	-	0.032252
XLOC_000405	-	0.034852
XLOC_000447	ELL3	0.038059
XLOC_000507	RASGRP1	0.002372
XLOC_000519	SCG5	0.002372
XLOC_000544	OTUD7A	0.025115
XLOC_000563	ENSSSCG00000004870	0.004177
XLOC_000579	ENSSSCG00000021694,SERPINB11	0.002372
XLOC_000581	-	0.002372
XLOC_000582	SERPINB2	0.010072
XLOC_000615	SMAD6	0.002372
XLOC_000643	TRAPPC6B	0.017207
XLOC_000690	LGALS3	0.002372

XLOC_000694	PELI2	0.002372
XLOC_000701	ENSSSCG00000022299	0.028943
XLOC_000716	PKC	0.008746
XLOC_000777	INTERFERON-BETA-1	0.002372
XLOC_000784	ADFP,ENSSSCG00000005172	0.002372
XLOC_000788	PSIP1	0.004177
XLOC_000789	TTC39B	0.002372
XLOC_000840	ENSSSCG00000020904	0.041848
XLOC_000841	ENSSSCG00000005260	0.005845
XLOC_000845	ANXA1	0.002372
XLOC_000858	PSAT1	0.002372
XLOC_000870	RUSC2	0.002372
XLOC_000902	TDRD7	0.004177
XLOC_000916	SEC61B	0.042609
XLOC_000947	FKTN	0.021314
XLOC_000950	ENSSSCG00000005431	0.045889
XLOC_000962	ENSSSCG00000005451	0.008746
XLOC_000966	GNG10	0.035659
XLOC_000970	KIAA1958	0.011350
XLOC_000976	ENSSSCG00000029221	0.002372
XLOC_000994	TLR4	0.002372
XLOC_001044	NEK6	0.008746
XLOC_001067	STXBP1	0.002372
XLOC_001074	C9orf16,LCN2	0.025115
XLOC_001082	SPTAN1	0.022312
XLOC_001097	TOR1B	0.002372
XLOC_001129	COL5A1	0.010072
XLOC_001130	FCN2	0.002372
XLOC_001131	OLFM1	0.005845
XLOC_001145	PARD6G	0.028943
XLOC_001204	THBS2	0.033125
XLOC_001212	AGPAT4	0.048740
XLOC_001230	DYNLT1	0.002372
XLOC_001231	SYTL3	0.002372
XLOC_001238	SNX9	0.033995
XLOC_001246	ENSSSCG00000004072	0.019332
XLOC_001247	ENSSSCG00000025352	0.022312
XLOC_001252	SYNE1	0.002372
XLOC_001258	ARMT1	0.028943
XLOC_001263	LRP11	0.049392
XLOC_001278	RAB32	0.011350
XLOC_001281	ENSSSCG00000004123	0.020320
XLOC_001282	-	0.002372

XLOC_001283	ENSSSCG00000026681	0.010072
XLOC_001285	STX11	0.002372
XLOC_001299	-	0.018305
XLOC_001305	TNFAIP3	0.002372
XLOC_001352	-	0.040229
XLOC_001361	SMPDL3A	0.002372
XLOC_001374	NUS1	0.005845
XLOC_001376	DCBLD1	0.004177
XLOC_001424	RRAGD	0.007395
XLOC_001429	-	0.002372
XLOC_001466	GL	0.033995
XLOC_001509	FAM26F	0.002372
XLOC_001541	PSTPIP2	0.002372
XLOC_001585	DAPK2	0.002372
XLOC_001617	AQP9	0.002372
XLOC_001632	MAPK6	0.008746
XLOC_001651	SEMA6D	0.002372
XLOC_001654	SLC28A2	0.002372
XLOC_001658	SORD	0.022312
XLOC_001675	ENSSSCG00000004705	0.010072
XLOC_001688	ENSSSCG00000020666	0.029810
XLOC_001690	ENSSSCG00000024969	0.024202
XLOC_001723	THBS1	0.002372
XLOC_001725	SPRED1	0.002372
XLOC_001784	-	0.002372
XLOC_001796	SERPINB8	0.002372
XLOC_001797	SERPINB10	0.002372
XLOC_001802	TNFRSF11A	0.004177
XLOC_001859	PNN	0.015000
XLOC_001892	GNG2	0.021314
XLOC_001933	ISG20	0.002372
XLOC_002028	MAMDC2	0.002372
XLOC_002033	-	0.043224
XLOC_002035	ENSSSCG00000024257	0.002372
XLOC_002062	SIT1	0.008746
XLOC_002063	Rse_MRP	0.004177
XLOC_002065	TPM2	0.048740
XLOC_002099	CORO2A,TBC1D2	0.010072
XLOC_002100	ENSSSCG00000005377,ENSSSCG00000027558	0.002372
XLOC_002121	ENSSSCG00000005423	0.029810
XLOC_002126	KLF4	0.002372
XLOC_002140	TRX1	0.028943
XLOC_002158	ENSSSCG00000005477	0.029810

XLOC_002163	RGS3	0.037305
XLOC_002175	TNFRSF8	0.029810
XLOC_002183	ENSSSCG00000028117	0.022312
XLOC_002184	MEGF9	0.002372
XLOC_002234	STRBP	0.023280
XLOC_002258	FAM129B	0.002372
XLOC_002290	DYT1	0.002372
XLOC_002312	SLC2A6	0.041848
XLOC_002320	FCN1	0.002372
XLOC_002417	-	0.015000
XLOC_002521	-	0.002372
XLOC_002611	-	0.027994
XLOC_002619	-	0.013835
XLOC_002631	-	0.002372
XLOC_002635	-	0.002372
XLOC_002640	-	0.026101
XLOC_002658	-	0.002372
XLOC_002685	-	0.010072
XLOC_002756	-	0.007395
XLOC_002776	-	0.041848
XLOC_002793	-	0.002372
XLOC_002849	-	0.002372
XLOC_002851	-	0.021314
XLOC_002863	-	0.029810
XLOC_002948	-	0.005845
XLOC_003092	-	0.004177
XLOC_003097	-	0.002372
XLOC_003118	-	0.004177
XLOC_003119	-	0.008746
XLOC_003141	-	0.033995
XLOC_003144	-	0.040229
XLOC_003328	-	0.013835
XLOC_003330	-	0.002372
XLOC_003347	-	0.042609
XLOC_003353	-	0.045290
XLOC_003355	-	0.027058
XLOC_003356	-	0.044653
XLOC_003492	-	0.007395
XLOC_003500	-	0.005845
XLOC_003508	-	0.002372
XLOC_003522	-	0.002372
XLOC_003532	-	0.002372
XLOC_003539	-	0.002372
XLOC_003606	-	0.002372

XLOC_003607	-	0.023280
XLOC_003609	-	0.007395
XLOC_003610	-	0.022312
XLOC_003639	-	0.034852
XLOC_003700	-	0.002372
XLOC_003794	-	0.026101
XLOC_003796	-	0.005845
XLOC_003811	-	0.002372
XLOC_003812	-	0.002372
XLOC_003846	-	0.011350
XLOC_003858	-	0.041848
XLOC_003926	-	0.048740
XLOC_003945	DEGS1	0.002372
XLOC_003946	CNIH4	0.002372
XLOC_003955	TMEM63A	0.002372
XLOC_003986	CFH	0.015000
XLOC_003991	DENND1B	0.002372
XLOC_004000	ENSSSCG00000010915	0.002372
XLOC_004001	-	0.008746
XLOC_004019	FBP1	0.002372
XLOC_004051	AQP3	0.002372
XLOC_004059	DDX58	0.042609
XLOC_004068	C9orf72	0.002372
XLOC_004089	TMEM236	0.016103
XLOC_004114	MPP7	0.011350
XLOC_004126	ARHGAP21	0.005845
XLOC_004168	ENSSSCG00000011129	0.002372
XLOC_004170	ENSSSCG00000021720,SFMBT2	0.045889
XLOC_004172	PRKCQ	0.013835
XLOC_004173	PFKFB3	0.002372
XLOC_004196	KLF6	0.027994
XLOC_004225	RGS1	0.030613
XLOC_004252	HLX	0.002372
XLOC_004283	EH	0.046618
XLOC_004285	PARP1	0.010072
XLOC_004316	ASPM	0.018305
XLOC_004323	-	0.021314
XLOC_004328	CAMSAP2	0.026101
XLOC_004340	ENSSSCG00000010913,V1	0.011350
XLOC_004342	V1	0.002372
XLOC_004365	DAPK1	0.031419
XLOC_004367	ENSSSCG00000010949	0.011350

XLOC_004382	SIGMAR1	0.027994
XLOC_004411	ENSSSCG00000011019,MAP3K8	0.002372
XLOC_004418	ENSSSCG00000020757	0.016103
XLOC_004458	ENSSSCG00000011076	0.002372
XLOC_004477	NRP1	0.002372
XLOC_004492	GATA3	0.002372
XLOC_004495	ENSSSCG00000024480	0.011350
XLOC_004606	-	0.023280
XLOC_004791	-	0.016103
XLOC_004808	-	0.005845
XLOC_004847	-	0.002372
XLOC_004866	-	0.002372
XLOC_004867	-	0.002372
XLOC_004871	-	0.002372
XLOC_004873	-	0.023280
XLOC_004885	-	0.045889
XLOC_004981	-	0.002372
XLOC_004983	-	0.012580
XLOC_005126	-	0.030613
XLOC_005174	SLC46A3	0.013835
XLOC_005184	ALOX5AP	0.002372
XLOC_005244	LPAR6	0.043224
XLOC_005250	KIAA0226L	0.008746
XLOC_005299	ENSSSCG00000009469	0.002372
XLOC_005325	DJC3	0.024202
XLOC_005356	TNFSF13B	0.015000
XLOC_005379	ENSSSCG00000009574	0.035659
XLOC_005401	ENSSSCG00000009293	0.018305
XLOC_005405	SHISA2	0.041076
XLOC_005456	-	0.031419
XLOC_005459	FOXO1	0.023280
XLOC_005472	CDADC1	0.002372
XLOC_005530	-	0.002372
XLOC_005537	-	0.027058
XLOC_005550	SPRY2	0.005845
XLOC_005623	RAB20	0.002372
XLOC_005624	-	0.018305
XLOC_005629	LAMP1	0.002372
XLOC_005637	GAS6	0.004177
XLOC_005662	-	0.036488
XLOC_005829	-	0.015000
XLOC_005841	-	0.002372
XLOC_005845	-	0.011350

XLOC_005862	-	0.027058
XLOC_006067	METRNL	0.002372
XLOC_006068	-	0.028943
XLOC_006072	SLC16A3	0.022312
XLOC_006090	TBC1D16	0.007395
XLOC_006094	TIMP-2	0.002372
XLOC_006097	-	0.002372
XLOC_006099	SCAR16	0.034852
XLOC_006109	RHBDF2	0.002372
XLOC_006124	GALK1	0.002372
XLOC_006141	CD300LF	0.005845
XLOC_006143	ENSSSCG00000020967	0.024202
XLOC_006144	CD300C	0.002372
XLOC_006148	-	0.020320
XLOC_006164	ARSG,SLC16A6	0.002372
XLOC_006206	PLEKHM1	0.018305
XLOC_006227	GRN	0.002372
XLOC_006286	CCR7	0.020320
XLOC_006289	TOP2A	0.007395
XLOC_006301	ENSSSCG00000017492	0.002372
XLOC_006320	TBX21	0.027994
XLOC_006461	CCL3L1	0.002372
XLOC_006465	CCL23	0.002372
XLOC_006466	CCL16	0.002372
XLOC_006467	CCL5	0.002372
XLOC_006484	CCL8	0.002372
XLOC_006485	CCL2	0.002372
XLOC_006506	ENSSSCG00000017754	0.002372
XLOC_006539	-	0.005845
XLOC_006581	SPNS3	0.011350
XLOC_006591	CAMTA2	0.015000
XLOC_006596	CXCL16	0.002372
XLOC_006631	ENSSSCG00000017962	0.004177
XLOC_006635	CHD3	0.002372
XLOC_006696	WDR45L	0.030613
XLOC_006700	-	0.033995
XLOC_006704	FASN	0.002372
XLOC_006705	ENSSSCG00000023044	0.007395
XLOC_006717	ENSSSCG00000017156	0.013835
XLOC_006732	PRCD	0.027994
XLOC_006734	SPHK1	0.005845
XLOC_006746	ITGB4	0.008746
XLOC_006766	-	0.016103
XLOC_006770	TTYH2	0.048079

XLOC_006793	AXIN2	0.008746
XLOC_006842	SLC4A1	0.031419
XLOC_006856	VAT1	0.002372
XLOC_006866	RAMP2	0.002372
XLOC_006872	HSD17B1,GLU	0.011350
XLOC_006902	KRT23	0.041076
XLOC_006907	RARA	0.008746
XLOC_006965	TMEM92	0.002372
XLOC_006993	SCPEP1	0.002372
XLOC_007043	SNORA11	0.027994
XLOC_007051	CCL4	0.002372
XLOC_007070	CDK5R1	0.025115
XLOC_007074	ADAP2	0.002372
XLOC_007085	WSB1	0.002372
XLOC_007086	NOS2	0.002372
XLOC_007113	ABHD15	0.045889
XLOC_007130	FAM101B	0.002372
XLOC_007135	RILP,SCARF1	0.020320
XLOC_007144	CLUH	0.035659
XLOC_007163	TAX1BP3	0.005845
XLOC_007164	ENSSSCG00000017868	0.045889
XLOC_007180	XAF1	0.002372
XLOC_007186	WSCD1	0.002372
XLOC_007198	PLD2	0.041848
XLOC_007200	ZMYND15	0.002372
XLOC_007210	BCL6B	0.002372
XLOC_007220	ENSSSCG00000025782	0.002372
XLOC_007344	-	0.002372
XLOC_007346	-	0.036488
XLOC_007347	-	0.002372
XLOC_007356	-	0.021314
XLOC_007445	-	0.004177
XLOC_007446	-	0.002372
XLOC_007486	-	0.002372
XLOC_007527	-	0.021314
XLOC_007626	-	0.002372
XLOC_007644	-	0.002372
XLOC_007658	-	0.002372
XLOC_007701	-	0.002372
XLOC_007702	-	0.002372
XLOC_007703	-	0.002372
XLOC_007706	-	0.008746
XLOC_007813	-	0.041076
XLOC_007814	-	0.038059

XLOC_007815	-	0.044653
XLOC_007854	-	0.016103
XLOC_007974	CCRL2	0.002372
XLOC_007978	PTH1R	0.002372
XLOC_008068	-	0.046618
XLOC_008114	BHLHE40	0.002372
XLOC_008154	ENSSSCG00000028582	0.027994
XLOC_008157	ENSSSCG00000011600	0.002372
XLOC_008183	-	0.002372
XLOC_008184	DJC13	0.021314
XLOC_008198	CEP63	0.013835
XLOC_008214	MRAS	0.024202
XLOC_008230	RASA2	0.007395
XLOC_008234	ENSSSCG00000011676	0.025115
XLOC_008251	RNF13	0.004177
XLOC_008261	P2RY1	0.021314
XLOC_008279	ENSSSCG00000027556	0.024202
XLOC_008283	MFSD1	0.002372
XLOC_008288	-	0.010072
XLOC_008299	-	0.002372
XLOC_008306	-	0.002372
XLOC_008309	-	0.047338
XLOC_008310	PKC	0.004177
XLOC_008396	-	0.013835
XLOC_008402	IL1RAP	0.002372
XLOC_008410	HES1	0.007395
XLOC_008438	OSBPL11	0.002372
XLOC_008445	-	0.044653
XLOC_008448	DIRC2	0.011350
XLOC_008466	-	0.004177
XLOC_008468	TMEM39A	0.024202
XLOC_008496	ENSSSCG00000011935	0.020320
XLOC_008506	-	0.002372
XLOC_008507	-	0.002372
XLOC_008510	-	0.002372
XLOC_008533	CLDND1	0.043224
XLOC_008556	GBE1	0.039463
XLOC_008562	ENSSSCG00000012006	0.002372
XLOC_008586	BACH1	0.013835
XLOC_008635	PFKL	0.026101
XLOC_008658	-	0.002372
XLOC_008660	-	0.002372
XLOC_008665	ANKRD28	0.002372
XLOC_008698	-	0.011350

XLOC_008737	CX3CR1	0.005845
XLOC_008772	CCR1	0.002372
XLOC_008774	LTF	0.002372
XLOC_008809	ENSSSCG00000011361	0.023280
XLOC_008812	SLC25A20	0.002372
XLOC_008826	UBA7	0.025115
XLOC_008833	HYAL2	0.019332
XLOC_008840	MAPKAPK3	0.002372
XLOC_008843	MANF	0.016103
XLOC_008861	ALAS1	0.010072
XLOC_008872	TKT	0.002372
XLOC_008885	ARHGEF3	0.002372
XLOC_008955	-	0.041848
XLOC_008984	SYN2	0.047338
XLOC_008995	ENSSSCG00000029810	0.020320
XLOC_009004	FBLN2	0.038059
XLOC_009030	ACPP	0.010072
XLOC_009109	P2RY13	0.002372
XLOC_009120	SLC33A1	0.010072
XLOC_009136	ENSSSCG00000027790	0.019332
XLOC_009175	ENSSSCG00000011752	0.002372
XLOC_009178	NCEH1	0.002372
XLOC_009185	-	0.002372
XLOC_009197	B3GNT5	0.011350
XLOC_009229	BCL6	0.005845
XLOC_009247	ATP13A3	0.034852
XLOC_009279	PDIA5	0.002372
XLOC_009282	ENSSSCG00000028575	0.039463
XLOC_009289	ENSSSCG00000027867	0.002372
XLOC_009295	EAF2	0.002372
XLOC_009306	CD80	0.002372
XLOC_009322	ATP6V1A,GRAMD1C	0.013835
XLOC_009342	TRAT1	0.008746
XLOC_009350	ALCAM	0.032252
XLOC_009355	SNORA18	0.020320
XLOC_009357	NFKBIZ,NXPE3	0.002372
XLOC_009383	GPR15	0.004177
XLOC_009401	CRYBG3,ENSSSCG00000022895	0.002372
XLOC_009414	ENSSSCG00000029384	0.041076
XLOC_009421	BTG3	0.002372
XLOC_009422	C21orf91	0.002372
XLOC_009454	TIAM1	0.030613
XLOC_009468	ENSSSCG00000024614	0.031419

XLOC_009489	-	0.048079
XLOC_009518	TRPM2	0.030613
XLOC_009535	LSS	0.007395
XLOC_009556	-	0.015000
XLOC_009559	-	0.048740
XLOC_009700	-	0.002372
XLOC_009825	-	0.029810
XLOC_009866	-	0.002372
XLOC_009885	-	0.002372
XLOC_010021	-	0.031419
XLOC_010279	-	0.004177
XLOC_010288	-	0.005845
XLOC_010462	-	0.047338
XLOC_010474	-	0.002372
XLOC_010590	-	0.043224
XLOC_010636	-	0.002372
XLOC_010667	-	0.002372
XLOC_010764	-	0.038749
XLOC_010848	-	0.016103
XLOC_010867	-	0.046618
XLOC_010879	-	0.002372
XLOC_010882	-	0.023280
XLOC_010883	-	0.034852
XLOC_010908	-	0.032252
XLOC_010913	-	0.002372
XLOC_010915	-	0.002372
XLOC_010917	-	0.016103
XLOC_010928	-	0.002372
XLOC_010935	-	0.002372
XLOC_010936	-	0.010072
XLOC_010969	-	0.023280
XLOC_010987	-	0.002372
XLOC_011035	DMTN	0.002372
XLOC_011044	ENSSSCG00000009627	0.002372
XLOC_011052	ADAM28	0.005845
XLOC_011081	ENSSSCG00000027568	0.029810
XLOC_011125	ENSSSCG00000009761	0.044005
XLOC_011141	ENSSSCG00000021261	0.002372
XLOC_011174	CCDC63	0.002372
XLOC_011178	SH2B3	0.011350
XLOC_011210	OAS2	0.002372
XLOC_011212	OAS1	0.046618
XLOC_011222	U4	0.024202
XLOC_011249	CMLKR1	0.002372

XLOC_011269	EMID1	0.002372
XLOC_011284	ENSSSCG00000010011	0.008746
XLOC_011321	-	0.035659
XLOC_011328	ADORA2A	0.002372
XLOC_011364	GP1BB,SEPT5	0.017207
XLOC_011373	ENSSSCG00000026176	0.004177
XLOC_011383	-	0.011350
XLOC_011394	-	0.042609
XLOC_011406	TTC13	0.002372
XLOC_011428	-	0.005845
XLOC_011452	-	0.002372
XLOC_011460	EIF4EBP2	0.032252
XLOC_011478	PLAU	0.002372
XLOC_011492	ENSSSCG00000010330	0.002372
XLOC_011565	STAMBPL1	0.035659
XLOC_011568	IFIT3	0.002372
XLOC_011570	IFIT5	0.012580
XLOC_011579	HHEX	0.002372
XLOC_011580	-	0.007395
XLOC_011589	SLC35G1	0.002372
XLOC_011599	ENSSSCG00000010498,ENTPD1	0.002372
XLOC_011628	SCD	0.008746
XLOC_011684	TCF7L2	0.002372
XLOC_011710	BAG3	0.038749
XLOC_011715	ENSSSCG00000010692	0.017207
XLOC_011724	GMFG	0.012580
XLOC_011766	-	0.020320
XLOC_011792	NFIL3	0.002372
XLOC_011794	SPTLC1	0.008746
XLOC_011796	SLC18A1	0.011350
XLOC_011819	ENTPD4	0.015000
XLOC_011851	CTSB	0.002372
XLOC_011871	PUS1	0.040229
XLOC_011916	P2RX4	0.002372
XLOC_011917	P2X7R	0.027058
XLOC_011918	-	0.015000
XLOC_011924	HVCN1	0.008746
XLOC_011962	ALDH2	0.020320
XLOC_011978	ENSSSCG00000009926	0.002372
XLOC_011998	-	0.002372
XLOC_012015	OSM	0.020320
XLOC_012029	PATZ1,PIK3IP1	0.049392
XLOC_012090	MTR	0.002372

XLOC_012096	ENSSSCG00000010149,ENSSSCG00000024830	0.002372
XLOC_012103	-	0.002372
XLOC_012117	ARV1	0.002372
XLOC_012172	-	0.002372
XLOC_012180	PRF1	0.002372
XLOC_012186	PSAP	0.018305
XLOC_012193	P4HA1	0.044005
XLOC_012221	TMEM254	0.002372
XLOC_012271	ALOX5	0.002372
XLOC_012281	SGMS1	0.002372
XLOC_012285	ANKRD22	0.002372
XLOC_012288	LIPA	0.002372
XLOC_012292	HTR7	0.002372
XLOC_012295	ENSSSCG00000010464	0.002372
XLOC_012301	ENSSSCG00000010476	0.023280
XLOC_012324	-	0.004177
XLOC_012337	SLC25A28	0.002372
XLOC_012389	-	0.021314
XLOC_012397	ABLIM1	0.002372
XLOC_012442	CHST15	0.002372
XLOC_012503	-	0.002372
XLOC_012504	-	0.042609
XLOC_012513	-	0.002372
XLOC_012514	-	0.002372
XLOC_012515	-	0.002372
XLOC_012551	-	0.033995
XLOC_012565	-	0.002372
XLOC_012575	-	0.002372
XLOC_012605	-	0.007395
XLOC_012648	-	0.002372
XLOC_012750	-	0.002372
XLOC_012788	-	0.002372
XLOC_012828	-	0.002372
XLOC_012835	-	0.002372
XLOC_012885	-	0.005845
XLOC_012886	-	0.002372
XLOC_012888	-	0.013835
XLOC_012889	-	0.004177
XLOC_013058	-	0.017207
XLOC_013096	-	0.002372
XLOC_013110	-	0.002372
XLOC_013186	-	0.002372
XLOC_013206	-	0.002372

XLOC_013248	CXCR4	0.002372
XLOC_013276	MARCO	0.002372
XLOC_013277	STEAP3	0.041076
XLOC_013283	BIN1	0.008746
XLOC_013294	ENSSSCG00000015738	0.008746
XLOC_013297	PTPN4	0.004177
XLOC_013301	RALB	0.011350
XLOC_013328	WWC2	0.004177
XLOC_013366	GSR	0.037305
XLOC_013378	HS6ST1	0.018305
XLOC_013394	G3PD	0.002372
XLOC_013400	DAPL1	0.044653
XLOC_013443	CYBRD1	0.012580
XLOC_013460	-	0.002372
XLOC_013517	ENSSSCG00000027157	0.002372
XLOC_013518	ENSSSCG00000016040	0.002372
XLOC_013547	PLCL1	0.002372
XLOC_013564	C-FLIP	0.039463
XLOC_013567	ENSSSCG00000028157	0.039463
XLOC_013583	FAM117B	0.002372
XLOC_013587	TP44	0.026101
XLOC_013606	PLEKHM3	0.002372
XLOC_013637	ENSSSCG00000016183	0.034852
XLOC_013641	NRAMP1	0.002372
XLOC_013670	RAB11FIP5	0.016103
XLOC_013693	-	0.013835
XLOC_013757	TRAF3IP1	0.044005
XLOC_013811	-	0.005845
XLOC_013823	SPOPL	0.008746
XLOC_013824	HNMT	0.002372
XLOC_013840	SLC35F5	0.007395
XLOC_013900	FACL2	0.002372
XLOC_013904	UFSP2	0.037305
XLOC_013916	ADAM-9	0.019332
XLOC_013933	PROSC	0.048079
XLOC_013942	ENSSSCG00000025008,NRG1	0.011350
XLOC_013957	PPP1R3B	0.011350
XLOC_013976	-	0.035659
XLOC_013981	ACVR1	0.044653
XLOC_014020	ENSSSCG00000022496,ENSSSCG00000023421	0.010072
XLOC_014077	ENSSSCG00000026081	0.002372
XLOC_014081	ENSSSCG00000027221	0.002372

XLOC_014090	STAT4	0.002372
XLOC_014097	GTF3C3	0.002372
XLOC_014123	ENSSSCG00000016106	0.004177
XLOC_014160	IDH1	0.002372
XLOC_014166	IKZF2	0.002372
XLOC_014170	FN1	0.002372
XLOC_014172	MREG	0.005845
XLOC_014179	ENSSSCG00000016184	0.002372
XLOC_014217	-	0.021314
XLOC_014237	DNER	0.020320
XLOC_014242	GPR55	0.004177
XLOC_014250	ENSSSCG00000016277	0.040229
XLOC_014263	-	0.004177
XLOC_014271	SCAR6	0.002372
XLOC_014365	-	0.004177
XLOC_014366	-	0.004177
XLOC_014373	-	0.004177
XLOC_014434	-	0.004177
XLOC_014436	-	0.002372
XLOC_014439	-	0.002372
XLOC_014444	-	0.002372
XLOC_014493	-	0.005845
XLOC_014496	-	0.002372
XLOC_014501	-	0.002372
XLOC_014506	-	0.002372
XLOC_014507	-	0.002372
XLOC_014525	-	0.044005
XLOC_014568	-	0.002372
XLOC_014637	-	0.002372
XLOC_014650	-	0.002372
XLOC_014651	-	0.010072
XLOC_014656	-	0.002372
XLOC_014701	-	0.002372
XLOC_014812	-	0.002372
XLOC_014829	-	0.002372
XLOC_014941	-	0.007395
XLOC_014979	-	0.018305
XLOC_015013	ENSSSCG00000029792	0.011350
XLOC_015015	ENSSSCG00000016781	0.002372
XLOC_015077	EMB,SNORD28	0.027994
XLOC_015088	GZMA	0.004177
XLOC_015102	-	0.007395
XLOC_015103	-	0.045889
XLOC_015127	LY64	0.002372

XLOC_015150	-	0.045290
XLOC_015154	DUSP1	0.010072
XLOC_015158	SH3PXD2B	0.002372
XLOC_015191	-	0.002372
XLOC_015204	ADAM19	0.002372
XLOC_015222	SLC36A1	0.002372
XLOC_015249	CH242-128B1.2	0.002372
XLOC_015262	ENSSSCG00000028803	0.012580
XLOC_015263	ENSSSCG00000025756	0.011350
XLOC_015288	-	0.037305
XLOC_015324	DAB2	0.008746
XLOC_015339	-	0.002372
XLOC_015377	PLK2	0.002372
XLOC_015436	-	0.002372
XLOC_015459	TENM2	0.004177
XLOC_015516	GM2A	0.045889
XLOC_015605	-	0.031419
XLOC_015682	-	0.025115
XLOC_015821	-	0.020320
XLOC_015823	-	0.002372
XLOC_015842	-	0.034852
XLOC_015856	-	0.002372
XLOC_015905	-	0.002372
XLOC_015968	-	0.002372
XLOC_016044	CSGALCT1	0.019332
XLOC_016047	ADAM3A	0.024202
XLOC_016050	AGPAT6	0.002372
XLOC_016059	AP3M2	0.005845
XLOC_016064	-	0.002372
XLOC_016120	ENSSSCG00000025340	0.025115
XLOC_016145	SIGLEC1	0.002372
XLOC_016198	ENSSSCG00000007239	0.002372
XLOC_016199	ENSSSCG00000007240	0.041848
XLOC_016200	-	0.007395
XLOC_016274	PPP1R16B	0.004177
XLOC_016302	SLPI	0.002372
XLOC_016314	MMP9	0.008746
XLOC_016343	C/EBP,CEBPB	0.002372
XLOC_016414	MSR1	0.002372
XLOC_016424	ASAH1	0.002372
XLOC_016434	IDO1	0.004177
XLOC_016459	PRP	0.002372
XLOC_016465	GPCPD1	0.002372
XLOC_016480	JAG1	0.002372

XLOC_016492	SNORD17	0.004177
XLOC_016513	CST3	0.011350
XLOC_016527	CDC25B	0.049392
XLOC_016531	BC1	0.039463
XLOC_016545	-	0.007395
XLOC_016546	TGM3	0.007395
XLOC_016573	ENSSSCG00000007228	0.036488
XLOC_016645	MAFB	0.002372
XLOC_016660	ADA	0.002372
XLOC_016666	ENSSSCG00000022258	0.002372
XLOC_016668	SDC4	0.002372
XLOC_016700	SULF2	0.002372
XLOC_016706	DRK1	0.043224
XLOC_016709	B4GALT5	0.002372
XLOC_016717	NFATC2	0.007395
XLOC_016719	ATP9A	0.027058
XLOC_016743	ZBP1	0.031419
XLOC_016750	CTSZ	0.045889
XLOC_016817	-	0.002372
XLOC_016820	-	0.002372
XLOC_016824	-	0.035659
XLOC_016825	-	0.013835
XLOC_016839	-	0.002372
XLOC_016841	-	0.004177
XLOC_016842	-	0.012580
XLOC_016908	-	0.004177
XLOC_016989	-	0.002372
XLOC_017025	-	0.045889
XLOC_017026	-	0.012580
XLOC_017107	-	0.002372
XLOC_017139	-	0.024202
XLOC_017140	-	0.015000
XLOC_017187	AOC1	0.022312
XLOC_017212	KIAA1147	0.025115
XLOC_017229	SVOPL	0.042609
XLOC_017233	CREB3L2	0.002372
XLOC_017271	KCP	0.035659
XLOC_017304	TSPAN12	0.002372
XLOC_017313	TFEC	0.002372
XLOC_017325	ENSSSCG00000016648,ENSSSCG00000025602	0.002372
XLOC_017352	INMT	0.029810
XLOC_017380	CYCS	0.017207
XLOC_017388	TNS3	0.002372

XLOC_017393	ENSSSCG00000016732,SNORA5	0.002372
XLOC_017416	PSMA2	0.004177
XLOC_017459	GALNTL5	0.004177
XLOC_017493	EPHB6,TRBC1,TRBV21	0.019332
XLOC_017499	ENSSSCG00000029386	0.013835
XLOC_017502	-	0.045290
XLOC_017504	SSBP1	0.026101
XLOC_017516	TBXAS1	0.002372
XLOC_017518	-	0.028943
XLOC_017583	CALU	0.002372
XLOC_017585	ENSSSCG00000016584,FAM71F2	0.002372
XLOC_017596	GPR37	0.002372
XLOC_017619	TES	0.010072
XLOC_017669	CHN2	0.045889
XLOC_017680	ENSSSCG00000023695	0.002372
XLOC_017682	SNX10	0.013835
XLOC_017702	OGDH	0.043224
XLOC_017931	-	0.002372
XLOC_018078	-	0.002372
XLOC_018079	-	0.002372
XLOC_018080	-	0.039463
XLOC_018104	-	0.019332
XLOC_018171	-	0.033995
XLOC_018189	-	0.002372
XLOC_018191	-	0.033125
XLOC_018192	-	0.031419
XLOC_018204	-	0.002372
XLOC_018270	TPCN2	0.045889
XLOC_018272	CPT1A	0.002372
XLOC_018278	ENSSSCG00000028501	0.002372
XLOC_018279	ENSSSCG00000012893	0.010072
XLOC_018344	BATF2	0.002372
XLOC_018346	ATG2A	0.012580
XLOC_018379	ENSSSCG00000022404	0.002372
XLOC_018400	FADS2	0.002372
XLOC_018432	MPEG1	0.002372
XLOC_018498	SPI1	0.002372
XLOC_018502	ACP2	0.004177
XLOC_018514	PHF21A	0.007395
XLOC_018565	LGR4	0.002372
XLOC_018566	CCDC34	0.045290

XLOC_018584	ENSSSCG00000013353,ENSSSCG00000025576	0.004177
XLOC_018585	ENSSSCG00000021688	0.002372
XLOC_018595	LDHA	0.010072
XLOC_018606	PIK3C2A	0.002372
XLOC_018610	PLEKHA7	0.019332
XLOC_018671	-	0.002372
XLOC_018691	IFI30	0.002372
XLOC_018699	-	0.013835
XLOC_018700	SLC27A1	0.034852
XLOC_018702	ENSSSCG00000027848	0.002372
XLOC_018703	ENSSSCG00000013880,ENSSSCG00000026095	0.005845
XLOC_018727	ENSSSCG00000024211	0.004177
XLOC_018731	EPHX3	0.010072
XLOC_018757	-	0.008746
XLOC_018786	JUNB	0.027994
XLOC_018794	MAN2B1	0.004177
XLOC_018843	C19orf38	0.018305
XLOC_018866	RETN	0.002372
XLOC_018867	-	0.002372
XLOC_018880	ADGRE1,ENSSSCG00000022228	0.002372
XLOC_018888	TNFSF9	0.002372
XLOC_018928	MFSD12	0.007395
XLOC_018931	G11	0.011350
XLOC_018951	DF,ELANE	0.004177
XLOC_018954	ARID3A	0.002372
XLOC_018991	RNF130	0.008746
XLOC_018999	ADAMTS2	0.002372
XLOC_019017	HK3	0.002372
XLOC_019061	ENSSSCG00000014121	0.005845
XLOC_019062	FAM151B	0.002372
XLOC_019073	VCAN	0.008746
XLOC_019114	FER	0.022312
XLOC_019122	CAMK4,STARD4	0.007395
XLOC_019155	SNX24	0.002372
XLOC_019218	TGFBI	0.015000
XLOC_019232	EGR1	0.002372
XLOC_019235	CTN1	0.002372
XLOC_019238	SNORA74	0.012580
XLOC_019297	ABLIM3	0.002372
XLOC_019307	PPARGC1B	0.011350
XLOC_019365	AP2A2	0.027994

XLOC_019369	ATGL	0.022312
XLOC_019372	TALDO1	0.046618
XLOC_019400	CPT1A	0.016103
XLOC_019405	TCIRG1	0.002372
XLOC_019446	CTSW	0.002372
XLOC_019463	CDC42EP2	0.002372
XLOC_019465	ENSSSCG00000012998	0.002372
XLOC_019480	PLCB3	0.029810
XLOC_019491	-	0.002372
XLOC_019503	U2	0.007395
XLOC_019524	DAGLA	0.010072
XLOC_019537	CD5	0.013835
XLOC_019538	SLC15A3	0.002372
XLOC_019543	MS4A8B	0.048740
XLOC_019570	FAM111A	0.008746
XLOC_019571	FAM111B	0.002372
XLOC_019585	CTNND1	0.002372
XLOC_019591	C1-INH	0.004177
XLOC_019641	ENSSSCG00000013235,LXRA	0.002372
XLOC_019652	-	0.038059
XLOC_019690	CD59	0.002372
XLOC_019697	PRRG4	0.002372
XLOC_019727	TMEM86A	0.004177
XLOC_019737	NUCB2	0.002372
XLOC_019753	-	0.002372
XLOC_019765	ADM	0.002372
XLOC_019802	-	0.002372
XLOC_019803	NLRP3	0.002372
XLOC_019818	MPV17L2	0.048740
XLOC_019825	B3GNT3	0.002372
XLOC_019847	ENSSSCG00000013842,ENSSSCG00000027903	0.002372
XLOC_019850	RASAL3	0.004177
XLOC_019880	ENSSSCG00000027610	0.002372
XLOC_019882	-	0.013835
XLOC_019892	CH242-184K22.3,IL27RA,RLN3	0.004177
XLOC_019908	DSE2	0.020320
XLOC_019920	ENSSSCG00000013716	0.038059
XLOC_019970	RAB3D	0.002372
XLOC_019971	TMEM205	0.008746
XLOC_020001	MCOLN1,PNPLA6	0.002372
XLOC_020010	-	0.002372

XLOC_020045	EBI3	0.021314
XLOC_020061	NCLN	0.012580
XLOC_020071	THOP1	0.002372
XLOC_020074	SPPL2B	0.008746
XLOC_020109	AZU1	0.005845
XLOC_020198	-	0.002372
XLOC_020227	KIAA0825	0.027058
XLOC_020237	LIX1	0.002372
XLOC_020251	ENSSSCG00000027074	0.027058
XLOC_020271	PGGT1B	0.025115
XLOC_020307	IRF1	0.002372
XLOC_020317	C5orf15	0.027058
XLOC_020320	TCF7	0.038059
XLOC_020360	-	0.032252
XLOC_020365	HBEGF	0.002372
XLOC_020370	CD14	0.002372
XLOC_020383	ARAP3	0.002372
XLOC_020417	-	0.002372
XLOC_020419	CSF1R,ENSSSCG00000021886	0.035659
XLOC_020514	-	0.002372
XLOC_020520	-	0.002372
XLOC_020551	-	0.007395
XLOC_020574	-	0.026101
XLOC_020637	-	0.023280
XLOC_020670	-	0.002372
XLOC_020688	-	0.033995
XLOC_020739	-	0.032252
XLOC_020808	-	0.002372
XLOC_020809	-	0.008746
XLOC_020810	-	0.012580
XLOC_020813	-	0.038059
XLOC_021002	-	0.016103
XLOC_021079	-	0.025115
XLOC_021126	-	0.002372
XLOC_021134	-	0.008746
XLOC_021137	-	0.007395
XLOC_021138	-	0.011350
XLOC_021140	-	0.002372
XLOC_021147	-	0.037305
XLOC_021200	-	0.002372
XLOC_021251	-	0.016103
XLOC_021280	-	0.029810
XLOC_021403	-	0.012580
XLOC_021412	ENSSSCG00000007548	0.002372

XLOC_021418	ENSSSCG00000007552	0.044653
XLOC_021488	SLC12A9	0.002372
XLOC_021497	ENSSSCG00000023912	0.002372
XLOC_021519	LAT2	0.021314
XLOC_021524	NCF1	0.012580
XLOC_021537	GUSB	0.002372
XLOC_021550	-	0.002372
XLOC_021554	TRIM72	0.002372
XLOC_021562	HSD3B7	0.042609
XLOC_021574	-	0.002372
XLOC_021586	SPNS1	0.002372
XLOC_021667	ROGDI	0.002372
XLOC_021687	MEFV	0.002372
XLOC_021693	-	0.017207
XLOC_021737	SLC9A3R2	0.002372
XLOC_021751	ATP6V0C	0.002372
XLOC_021779	ENSSSCG00000008087	0.002372
XLOC_021783	ENSSSCG00000022493	0.002372
XLOC_021794	MAL	0.016103
XLOC_021805	ASTL,DUSP2	0.002372
XLOC_021823	IL1R2	0.002372
XLOC_021824	-	0.002372
XLOC_021829	RNF149	0.027994
XLOC_021831	TBC1D8	0.002372
XLOC_021845	ENSSSCG00000030232,KIAA1211L	0.002372
XLOC_021859	-	0.010072
XLOC_021868	CD8B	0.027994
XLOC_021882	NKL	0.002372
XLOC_021958	-	0.030613
XLOC_021966	RAB1B	0.002372
XLOC_022006	CCDC88A	0.002372
XLOC_022009	ENSSSCG00000008409,RTN4	0.045889
XLOC_022057	MAP4K3	0.028943
XLOC_022158	ENSSSCG00000030115	0.043224
XLOC_022172	FAM49A	0.002372
XLOC_022195	CMPK2	0.027994
XLOC_022241	GRIFIN,LFNG	0.002372
XLOC_022242	CHST12	0.002372
XLOC_022265	ARPC1A	0.031419
XLOC_022301	-	0.007395
XLOC_022337	TPST1	0.002372
XLOC_022354	-	0.010072

XLOC_022357	ENSSSCG00000007758	0.002372
XLOC_022358	ITGAM	0.016103
XLOC_022373	ZNF768	0.002372
XLOC_022443	ABCC1	0.032252
XLOC_022452	LITAF	0.007395
XLOC_022504	ZNF205	0.027058
XLOC_022590	IL1B	0.002372
XLOC_022592	IL1A	0.002372
XLOC_022634	FHL2	0.037305
XLOC_022644	IL1RL1	0.002372
XLOC_022647	IL1R2	0.002372
XLOC_022648	MAP4K4	0.024202
XLOC_022660	INPP4A	0.015000
XLOC_022675	RPIA	0.017207
XLOC_022689	CD8A	0.002372
XLOC_022692	REEP1	0.005845
XLOC_022705	CAPG	0.013835
XLOC_022725	HK2	0.002372
XLOC_022761	GK	0.002372
XLOC_022770	MXD1	0.008746
XLOC_022773	ANXA4	0.002372
XLOC_022786	PLEK	0.002372
XLOC_022792	ENSSSCG00000027527	0.022312
XLOC_022827	-	0.013835
XLOC_022845	MSH2	0.038059
XLOC_022876	ENSSSCG00000008476	0.037305
XLOC_022893	ENSSSCG00000008491	0.002372
XLOC_022910	-	0.015000
XLOC_022955	SLC35F6	0.005845
XLOC_022961	RAB10	0.012580
XLOC_022975	RHOB	0.049392
XLOC_022990	-	0.012580
XLOC_022991	ENSSSCG00000008624	0.023280
XLOC_023061	-	0.047338
XLOC_023161	-	0.004177
XLOC_023293	-	0.002372
XLOC_023300	-	0.002372
XLOC_023319	-	0.002372
XLOC_023320	-	0.043224
XLOC_023321	-	0.036488
XLOC_023416	-	0.042609
XLOC_023419	-	0.040229
XLOC_023433	-	0.002372
XLOC_023437	-	0.012580

XLOC_023451	-	0.002372
XLOC_023453	-	0.047338
XLOC_023481	-	0.002372
XLOC_023500	-	0.032252
XLOC_023501	-	0.002372
XLOC_023509	-	0.042609
XLOC_023669	-	0.002372
XLOC_023674	-	0.002372
XLOC_023728	-	0.045889
XLOC_023729	-	0.002372
XLOC_023743	-	0.004177
XLOC_023851	-	0.033995
XLOC_023861	-	0.008746
XLOC_023980	-	0.032252
XLOC_023981	-	0.026101
XLOC_024017	-	0.004177
XLOC_024018	-	0.002372
XLOC_024041	-	0.044005
XLOC_024107	SLC45A4	0.002372
XLOC_024117	ssc-mir-30d	0.012580
XLOC_024170	EXT1	0.004177
XLOC_024186	TMEM74	0.019332
XLOC_024205	NCALD	0.002372
XLOC_024226	STK3	0.018305
XLOC_024227	-	0.004177
XLOC_024313	SNORD87	0.002372
XLOC_024327	GGH	0.002372
XLOC_024336	TOX	0.011350
XLOC_024350	ATP6V1H	0.002372
XLOC_024371	SLC19A2	0.002372
XLOC_024378	MPC2	0.024202
XLOC_024381	CD3Z	0.002372
XLOC_024416	LY9	0.002372
XLOC_024422	PEX19	0.048079
XLOC_024425	SLAMF9	0.007395
XLOC_024461	FCRL3	0.005845
XLOC_024482	GLMP	0.002372
XLOC_024501	GBA	0.007395
XLOC_024525	SLC39A1	0.019332
XLOC_024531	S100A9	0.002372
XLOC_024540	S100A11	0.005845
XLOC_024565	ARNT,CTSK,CTSS	0.008746
XLOC_024593	PRKAB2	0.002372
XLOC_024605	ZNF697	0.002372

XLOC_024626	TSPAN2	0.002372
XLOC_024639	SLC16A1	0.002372
XLOC_024668	PSMA5	0.019332
XLOC_024711	F3	0.002372
XLOC_024720	ENSSSCG00000006901	0.030613
XLOC_024743	GTF2B	0.041848
XLOC_024780	GSDMD	0.002372
XLOC_024793	ENSSSCG00000005941	0.020320
XLOC_024810	SQLE	0.033125
XLOC_024831	DEPTOR	0.012580
XLOC_024838	-	0.005845
XLOC_024842	AARD	0.002372
XLOC_024874	ATP6V1C1	0.004177
XLOC_024876	KLF10	0.004177
XLOC_024898	PGCP	0.002372
XLOC_024900	SDC2	0.002372
XLOC_024954	LY96	0.015000
XLOC_024974	ADHFE1	0.010072
XLOC_024975	RRS1	0.038059
XLOC_025004	MRPL15	0.031419
XLOC_025018	BLZF1	0.033125
XLOC_025019	ATP1B1	0.002372
XLOC_025048	UAP1	0.036488
XLOC_025050	SH2D1B	0.018305
XLOC_025058	CD32	0.007395
XLOC_025063	FCER1G,NDUFS2	0.041076
XLOC_025114	PCD1B	0.002372
XLOC_025121	-	0.008746
XLOC_025141	LM	0.002372
XLOC_025144	LAMTOR2	0.002372
XLOC_025161	ENSSSCG00000006533	0.022312
XLOC_025193	S100A12	0.002372
XLOC_025194	S100A8	0.002372
XLOC_025212	PIP5K1A	0.011350
XLOC_025214	TNFAIP8L2	0.002372
XLOC_025222	ADAMTSL4	0.002372
XLOC_025223	ECM1	0.002372
XLOC_025263	ENSSSCG00000030938,PHGDH	0.002372
XLOC_025281	CD2	0.002372
XLOC_025283	ATP1A1	0.030613
XLOC_025284	MAB21L3,SLC22A15	0.008746
XLOC_025294	-	0.042609
XLOC_025343	FAM102B	0.024202
XLOC_025344	-	0.002372

XLOC_025359	VCAM1	0.046618
XLOC_025368	ENSSSCG00000006877	0.027058
XLOC_025556	-	0.038749
XLOC_025616	-	0.008746
XLOC_025617	-	0.047338
XLOC_025619	-	0.013835
XLOC_025620	-	0.039463
XLOC_025628	-	0.038749
XLOC_025678	-	0.004177
XLOC_025722	-	0.037305
XLOC_025727	-	0.020320
XLOC_025729	-	0.002372
XLOC_025730	-	0.023280
XLOC_025829	-	0.018305
XLOC_025830	-	0.012580
XLOC_025832	-	0.005845
XLOC_025833	-	0.045290
XLOC_025848	-	0.010072
XLOC_025915	-	0.020320
XLOC_025956	-	0.002372
XLOC_025960	-	0.002372
XLOC_025962	-	0.002372
XLOC_025963	-	0.002372
XLOC_026020	-	0.027994
XLOC_026025	-	0.027994
XLOC_026034	-	0.002372
XLOC_026117	-	0.002372
XLOC_026120	-	0.002372
XLOC_026121	-	0.012580
XLOC_026318	ENSSSCG00000000018	0.013835
XLOC_026339	-	0.019332
XLOC_026351	SLC25A17	0.002372
XLOC_026397	ENSSSCG00000029668	0.002372
XLOC_026458	-	0.002372
XLOC_026459	ACVRL1	0.002372
XLOC_026460	ACVR1B	0.002372
XLOC_026468	CH242-185F9.2	0.002372
XLOC_026477	SPRYD3	0.022312
XLOC_026576	ENSSSCG00000000423	0.010072
XLOC_026589	SLC16A7	0.002372
XLOC_026616	IFN-GAMMA	0.018305
XLOC_026652	FGD4	0.002372
XLOC_026682	-	0.027058
XLOC_026710	-	0.020320

XLOC_026712	ENSSSCG00000030611	0.004177
XLOC_026717	LRP6	0.002372
XLOC_026727	ENSSSCG00000000640	0.002372
XLOC_026728	KLRK1	0.002372
XLOC_026737	CLEC2B	0.002372
XLOC_026738	CD69	0.016103
XLOC_026739	CLECL1	0.016103
XLOC_026741	KLRB1	0.002372
XLOC_026744	A2M	0.016103
XLOC_026746	KLRG1	0.002372
XLOC_026750	SLC2A3	0.002372
XLOC_026753	ENSSSCG00000026009	0.002372
XLOC_026767	TNFRSF1A	0.007395
XLOC_026803	USP18	0.011350
XLOC_026848	TXNRD1	0.002372
XLOC_026893	LTA4H	0.005845
XLOC_026903	PLXNC1	0.031419
XLOC_026921	ATP2B1	0.008746
XLOC_026945	DYRK2	0.017207
XLOC_026981	TSPO	0.017207
XLOC_026991	-	0.002372
XLOC_027021	MAFF	0.005845
XLOC_027026	ENSSSCG00000025720	0.031419
XLOC_027037	CYTH4	0.021314
XLOC_027040	CSF2RB	0.002372
XLOC_027041	NCF4	0.004177
XLOC_027073	-	0.002372
XLOC_027113	ENSSSCG00000000246	0.002372
XLOC_027126	RARG	0.004177
XLOC_027144	NFE2	0.028943
XLOC_027145	ENSSSCG00000000291	0.002372
XLOC_027146	ZNF385A	0.017207
XLOC_027179	CD63	0.004177
XLOC_027181	MMP19	0.002372
XLOC_027204	APON,TIMELESS	0.024202
XLOC_027222	ENSSSCG00000023150	0.045290
XLOC_027224	ENSSSCG00000023150	0.002372
XLOC_027240	LRIG3	0.005845
XLOC_027246	ENSSSCG00000023715	0.002372
XLOC_027256	-	0.011350
XLOC_027268	LYZ	0.002372
XLOC_027310	STK38L	0.002372
XLOC_027326	GOLT1B	0.002372
XLOC_027344	ENSSSCG00000000600	0.002372

XLOC_027350	PLBD1	0.002372
XLOC_027353	HEBP1	0.002372
XLOC_027356	BCL2L14	0.029810
XLOC_027362	-	0.027058
XLOC_027378	ENSSSCG00000025680	0.002372
XLOC_027404	LTBR	0.004177
XLOC_027416	TIGAR	0.032252
XLOC_027427	ENSSSCG00000000744,FKBP4	0.012580
XLOC_027505	DRAM1	0.002372
XLOC_027506	CHPT1	0.002372
XLOC_027554	ENSSSCG00000000921	0.002372
XLOC_027568	ACSS3	0.002372
XLOC_027606	-	0.023280
XLOC_027611	-	0.044653
XLOC_027653	-	0.048079
XLOC_027682	-	0.049392
XLOC_027690	-	0.017207
XLOC_027928	-	0.002372
XLOC_027930	-	0.002372
XLOC_027936	-	0.002372
XLOC_027944	-	0.010072
XLOC_027962	-	0.028943
XLOC_027964	-	0.002372
XLOC_027968	-	0.002372
XLOC_028092	-	0.016103
XLOC_028159	-	0.016103
XLOC_028284	-	0.002372
XLOC_028321	ADGRG5	0.005845
XLOC_028322	GPR56	0.002372
XLOC_028323	ADGRG3	0.002372
XLOC_028353	NFATC3	0.038059
XLOC_028380	ENSSSCG00000026318	0.002372
XLOC_028433	ZBTB32	0.016103
XLOC_028464	SPINT2	0.002372
XLOC_028496	ITPKC	0.022312
XLOC_028538	ENSSSCG00000003064	0.041848
XLOC_028575	C5AR1	0.002372
XLOC_028576	C5AR2,DHX34	0.002372
XLOC_028603	PPP1R15A	0.002372
XLOC_028622	ADM5,CPT1C	0.033995
XLOC_028625	AP2A1	0.002372
XLOC_028630	ENSSSCG00000003202	0.002372
XLOC_028645	ENSSSCG00000003226	0.002372
XLOC_028680	-	0.008746

XLOC_028688	-	0.002372
XLOC_028698	ENSSSCG00000029500	0.004177
XLOC_028820	PGD	0.002372
XLOC_028835	PLOD1	0.002372
XLOC_028839	-	0.002372
XLOC_028854	TMEM51	0.011350
XLOC_028868	PADI4	0.002372
XLOC_028870	ENSSSCG00000022946	0.013835
XLOC_028885	CDA	0.002372
XLOC_028898	C1QA	0.002372
XLOC_028899	C1QC	0.002372
XLOC_028900	C1QB	0.040229
XLOC_028907	CNR2,FUCA1	0.025115
XLOC_028913	RCAN3	0.048079
XLOC_028932	HMGN2	0.027994
XLOC_028950	STX12	0.024202
XLOC_028953	THEMIS2	0.043224
XLOC_028954	SMPDL3B	0.033125
XLOC_028972	SDC3	0.002372
XLOC_029015	UTP11L	0.002372
XLOC_029041	RAB31	0.019332
XLOC_029093	TTR	0.002372
XLOC_029117	MCOLN2	0.042609
XLOC_029120	PRKACG	0.039463
XLOC_029148	GPR177	0.002372
XLOC_029154	PDE4B	0.004177
XLOC_029179	DHCR24	0.012580
XLOC_029199	ZFYVE9	0.011350
XLOC_029276	P3H1	0.048079
XLOC_029292	VPS9D1,ZNF276	0.004177
XLOC_029322	KIAA0513	0.002372
XLOC_029354	MLKL	0.008746
XLOC_029359	ENSSSCG00000002723,EXOSC6	0.038749
XLOC_029377	ENSSSCG00000002743	0.034852
XLOC_029400	CCDC102A	0.005845
XLOC_029426	ATP6V0D1	0.005845
XLOC_029437	DUS2	0.004177
XLOC_029447	MMP2	0.008746
XLOC_029453	ENSSSCG00000002843	0.018305
XLOC_029472	C/EBP	0.002372
XLOC_029478	GPI	0.005845
XLOC_029484	FFAR2	0.002372
XLOC_029498	NFKBID	0.026101

XLOC_029499	TYROBP	0.002372
XLOC_029525	RASGRP4	0.005845
XLOC_029565	PLD3	0.004177
XLOC_029570	AXL	0.002372
XLOC_029592	ETHE1	0.002372
XLOC_029597	PLAUR	0.002372
XLOC_029610	PVR	0.022312
XLOC_029617	PGLYRP	0.004177
XLOC_029619	PTGIR	0.002372
XLOC_029622	PRKD2	0.019332
XLOC_029625	AP2S1	0.007395
XLOC_029708	ENSSSCG00000024518,SIGLEC5	0.002372
XLOC_029722	NLRP12	0.002372
XLOC_029727	TARM1	0.002372
XLOC_029728	OSCAR	0.030613
XLOC_029732	MBOAT7	0.007395
XLOC_029734	ENSSSCG00000003272	0.002372
XLOC_029735	ENSSSCG00000025549	0.030613
XLOC_029898	PADI2	0.002372
XLOC_029900	ARHGEF10L	0.002372
XLOC_029906	AKR7A2	0.004177
XLOC_029922	ECE1	0.002372
XLOC_029937	TCEB3	0.010072
XLOC_029943	RUNX3	0.002372
XLOC_029944	-	0.002372
XLOC_029965	FGR	0.002372
XLOC_030013	RNF19B	0.002372
XLOC_030014	AZIN2	0.008746
XLOC_030019	ENSSSCG00000003626	0.002372
XLOC_030054	MYCL	0.002372
XLOC_030071	-	0.042609
XLOC_030080	EPB41L3	0.002372
XLOC_030085	EMILIN2	0.031419
XLOC_030087	ENSSSCG00000027264	0.039463
XLOC_030107	NPC1	0.002372
XLOC_030143	ENSSSCG00000025209	0.005845
XLOC_030148	PRKACB	0.024202
XLOC_030151	ADGRL2	0.002372
XLOC_030153	IFI44	0.002372
XLOC_030167	ACADM	0.036488
XLOC_030181	SLC35D1	0.030613
XLOC_030183	ENSSSCG00000030423	0.002372
XLOC_030233	ZYG11A	0.015000

XLOC_030245	SPATA6	0.048079
XLOC_030375	-	0.005845
XLOC_030563	-	0.026101
XLOC_030600	-	0.002372
XLOC_030709	-	0.046618
XLOC_030739	-	0.002372
XLOC_030752	-	0.002372
XLOC_030806	-	0.004177
XLOC_030866	-	0.017207
XLOC_030951	-	0.026101
XLOC_030952	-	0.023280
XLOC_030962	-	0.021314
XLOC_030980	-	0.017207
XLOC_030982	-	0.002372
XLOC_031021	-	0.039463
XLOC_031046	-	0.002372
XLOC_031081	-	0.008746
XLOC_031145	-	0.002372
XLOC_031146	-	0.002372
XLOC_031235	-	0.028943
XLOC_031345	-	0.007395
XLOC_031394	-	0.038749
XLOC_031415	-	0.023280
XLOC_031468	EDN1	0.002372
XLOC_031469	-	0.022312
XLOC_031472	CD83	0.004177
XLOC_031493	SOX4	0.004177
XLOC_031559	Metazoa_SRP	0.005845
XLOC_031575	UBD	0.002372
XLOC_031627	LTB	0.007395
XLOC_031632	SLA-6	0.032252
XLOC_031680	ANKS1A	0.002372
XLOC_031726	-	0.017207
XLOC_031758	SLC29A1	0.019332
XLOC_031804	FAH	0.002372
XLOC_031806	ABHD17C	0.002372
XLOC_031808	MESDC2	0.005845
XLOC_031831	FURIN	0.028943
XLOC_031832	FES	0.005845
XLOC_031854	ZNF710	0.017207
XLOC_031876	SCAMP2	0.020320
XLOC_031881	-	0.004177
XLOC_031889	HEXA	0.002372
XLOC_031892	PKM	0.005845

XLOC_031904	NFKBIA	0.002372
XLOC_031914	EAPP	0.046618
XLOC_031917	U6	0.045889
XLOC_031924	HEATR5A	0.028943
XLOC_031933	GZMH	0.007395
XLOC_031949	JPH4	0.018305
XLOC_031960	CEBPE	0.022312
XLOC_031963	ENSSSCG00000002036	0.020320
XLOC_032047	ENSSSCG00000022551	0.027058
XLOC_032057	HSPA2	0.015000
XLOC_032089	SIPA1L1	0.048740
XLOC_032093	PSEN1	0.002372
XLOC_032113	FOS	0.002372
XLOC_032122	VASH1	0.008746
XLOC_032151	GPR65	0.040229
XLOC_032159	KCNK13	0.002372
XLOC_032164	SLC24A4	0.023280
XLOC_032271	SERPINB1	0.002372
XLOC_032284	-	0.018305
XLOC_032314	ENSSSCG00000020858	0.002372
XLOC_032321	MBOAT1	0.002372
XLOC_032449	IER3	0.002372
XLOC_032452	SUSC-MIC2	0.021314
XLOC_032460	AIF1	0.002372
XLOC_032462	LST1	0.002372
XLOC_032464	TNFA	0.002372
XLOC_032474	BF,C2	0.005845
XLOC_032561	FKBP5	0.019332
XLOC_032596	TREM-1	0.011350
XLOC_032632	NFKBIE	0.002372
XLOC_032647	TNFRSF21	0.002372
XLOC_032682	CTSH	0.008746
XLOC_032687	BCL2A1	0.002372
XLOC_032732	C7H15orf38	0.002372
XLOC_032758	SCAMP5	0.005845
XLOC_032787	-	0.002372
XLOC_032809	GZMB,GZMH	0.007395
XLOC_032812	ENSSSCG00000028443,SNORA11	0.015000
XLOC_032831	HOMEZ	0.022312
XLOC_032842	MMP14	0.019332
XLOC_032851	TRDC	0.002372
XLOC_032857	TRADV8	0.002372
XLOC_032874	ENSSSCG00000002084	0.038059

XLOC_032890	-	0.016103
XLOC_032937	ARRDC4	0.002372
XLOC_032940	-	0.002372
XLOC_032943	SLCO3A1	0.004177
XLOC_032950	SPTB	0.002372
XLOC_032958	ATP6V1D	0.002372
XLOC_032965	ZFP36L1	0.002372
XLOC_032985	ENSSSCG00000022178	0.002372
XLOC_032998	NPC2	0.024202
XLOC_033008	-	0.002372
XLOC_033027	GTF2A1	0.023280
XLOC_033030	-	0.038749
XLOC_033033	GALC	0.002372
XLOC_033052	TC2N	0.016103
XLOC_033055	ATXN3	0.025115
XLOC_033059	ENSSSCG00000002452	0.002372
XLOC_033087	BCL11B	0.002372
XLOC_033238	-	0.002372
XLOC_033240	-	0.002372
XLOC_033241	-	0.002372
XLOC_033243	-	0.002372
XLOC_033244	-	0.002372
XLOC_033245	-	0.022312
XLOC_033246	-	0.002372
XLOC_033250	-	0.002372
XLOC_033257	-	0.035659
XLOC_033275	-	0.002372
XLOC_033277	-	0.002372
XLOC_033359	-	0.002372
XLOC_033496	-	0.044005
XLOC_033542	-	0.002372
XLOC_033543	-	0.002372
XLOC_033544	-	0.007395
XLOC_033562	-	0.002372
XLOC_033657	-	0.002372
XLOC_033658	-	0.019332
XLOC_033661	-	0.002372
XLOC_033715	-	0.002372
XLOC_033718	-	0.002372
XLOC_033719	-	0.002372
XLOC_033857	-	0.002372
XLOC_033978	-	0.002372
XLOC_034000	-	0.025115
XLOC_034058	SH3BP2	0.007395

XLOC_034064	LREAP1	0.002372
XLOC_034070	JAKMIP1	0.010072
XLOC_034114	RBPJ	0.011350
XLOC_034124	ENSSSCG00000008769	0.002372
XLOC_034151	-	0.002372
XLOC_034206	TMEM144	0.002372
XLOC_034226	HOPX	0.002372
XLOC_034259	UTP3	0.048740
XLOC_034262	DCK	0.038749
XLOC_034263	SLC4A4	0.002372
XLOC_034272	CXCL8	0.002372
XLOC_034276	CXCL2,ENSSSCG00000008954	0.002372
XLOC_034278	EREG	0.023280
XLOC_034279	AREG	0.002372
XLOC_034285	-	0.002372
XLOC_034293	U1	0.021314
XLOC_034302	CCNG2	0.002372
XLOC_034351	ELMOD2	0.018305
XLOC_034353	CLGN	0.010072
XLOC_034376	TRPC3	0.002372
XLOC_034380	ANXA5	0.002372
XLOC_034407	ALPK1	0.032252
XLOC_034419	LEF1	0.043224
XLOC_034474	FAM13A	0.002372
XLOC_034481	HSD17B11	0.002372
XLOC_034522	MFSD7	0.002372
XLOC_034578	TAPT1	0.018305
XLOC_034584	ENSSSCG00000023604	0.028943
XLOC_034585	-	0.027058
XLOC_034608	ARAP2	0.019332
XLOC_034612	TLR1,TLR6	0.005845
XLOC_034624	RBM47	0.002372
XLOC_034631	ENSSSCG00000008803	0.034852
XLOC_034688	RAPGEF2	0.008746
XLOC_034746	JCHAIN	0.002372
XLOC_034748	-	0.018305
XLOC_034758	ALB	0.002372
XLOC_034770	AA	0.002372
XLOC_034776	SCARB2	0.004177
XLOC_034783	-	0.025115
XLOC_034795	TLR2	0.002372
XLOC_034834	-	0.002372
XLOC_034836	-	0.032252
XLOC_034837	ENSSSCG00000009057	0.002372

XLOC_034895	CCDC109B	0.002372
XLOC_034902	OSTC	0.010072
XLOC_034922	-	0.008746
XLOC_034923	SLC39A8	0.002372
XLOC_034953	ENSSSCG00000030548	0.002372
XLOC_034964	ENSSSCG00000009226	0.004177
XLOC_034968	PTPN13	0.002372
XLOC_034972	CDS1	0.002372
XLOC_034973	AGPAT9	0.002372
XLOC_035146	-	0.002372
XLOC_035313	-	0.034852
XLOC_035431	-	0.017207
XLOC_035442	-	0.037305
XLOC_035460	-	0.004177
XLOC_035461	-	0.002372
XLOC_035463	-	0.002372
XLOC_035555	-	0.030613
XLOC_035556	-	0.005845
XLOC_035594	-	0.008746
XLOC_035697	-	0.002372
XLOC_035708	DENND5A	0.038749
XLOC_035741	TPP1	0.045889
XLOC_035748	FAM160A2	0.015000
XLOC_035820	FOLR1	0.002372
XLOC_035875	PRCP	0.016103
XLOC_035885	-	0.002372
XLOC_035926	C11orf54	0.002372
XLOC_035970	RAB39A	0.002372
XLOC_035981	ZC3H12C	0.002372
XLOC_036011	CADM1	0.004177
XLOC_036025	IL10RA	0.002372
XLOC_036027	CD3E	0.002372
XLOC_036041	C2CD2L	0.002372
XLOC_036050	OAF	0.002372
XLOC_036057	SC5D	0.005845
XLOC_036082	VWA5A	0.002372
XLOC_036120	ETS1	0.025115
XLOC_036152	BTG2	0.002372
XLOC_036164	TMCC2	0.002372
XLOC_036169	RAB7B	0.002372
XLOC_036170	-	0.002372
XLOC_036171	C1orf186	0.002372
XLOC_036179	-	0.020320

XLOC_036189	C4BPA,ENSSSCG00000015663	0.002372
XLOC_036194	ENSSSCG00000015294	0.022312
XLOC_036205	CDK14	0.018305
XLOC_036208	GATAD1	0.011350
XLOC_036228	ENSSSCG00000015335	0.002372
XLOC_036248	ARL4A	0.002372
XLOC_036253	-	0.002372
XLOC_036262	ITGB8	0.017207
XLOC_036265	SP4	0.019332
XLOC_036272	IGF2BP3	0.029810
XLOC_036293	HGF	0.002372
XLOC_036296	GT3	0.033125
XLOC_036316	-	0.005845
XLOC_036375	ENSSSCG00000015499	0.007395
XLOC_036382	ENSSSCG00000023839	0.015000
XLOC_036432	PLA2G4A	0.002372
XLOC_036443	ATF3	0.002372
XLOC_036457	G0S2	0.002372
XLOC_036528	PRKCDBP	0.005845
XLOC_036607	OR51E1	0.002372
XLOC_036641	ARAP1	0.043224
XLOC_036683	PAK1	0.008746
XLOC_036694	GAB2	0.002372
XLOC_036707	SYTL2	0.002372
XLOC_036711	PICALM	0.022312
XLOC_036717	FZD4	0.002372
XLOC_036755	-	0.038059
XLOC_036759	MMP8	0.007395
XLOC_036773	CASP1,ENSSSCG00000014997	0.002372
XLOC_036790	RDX	0.041848
XLOC_036925	ARHGAP32	0.002372
XLOC_036948	SOX13	0.015000
XLOC_036960	NUAK2	0.012580
XLOC_036966	SLC45A3	0.002372
XLOC_036979	IL10	0.002372
XLOC_036980	-	0.025115
XLOC_037017	TFPI2	0.002372
XLOC_037026	PON2	0.013835
XLOC_037040	ETV1	0.002372
XLOC_037047	ENSSSCG00000029648	0.048079
XLOC_037051	TSPAN13	0.002372
XLOC_037093	ENSSSCG00000025166	0.041076
XLOC_037121	-	0.043224

XLOC_037122	ENSSSCG00000023584,ENSSSCG00000026913,ENSSSCG00000029596,TRGV11	0.038749
XLOC_037149	CHI3L1	0.002372
XLOC_037150	CHIT1	0.015000
XLOC_037156	FASLG	0.016103
XLOC_037172	RFWD2	0.010072
XLOC_037196	XPR1	0.002372
XLOC_037205	GLUL	0.004177
XLOC_037214	NCF2	0.002372
XLOC_037226	PTGS2	0.002372
XLOC_037229	CENPF	0.041848
XLOC_037235	FLVCR1	0.045290
XLOC_037259	-	0.044005
XLOC_037274	SEC61G	0.012580
XLOC_037293	-	0.002372
XLOC_037330	-	0.044005
XLOC_037350	-	0.013835
XLOC_037438	-	0.015000
XLOC_037456	-	0.002372
XLOC_037564	-	0.023280
XLOC_037590	-	0.002372
XLOC_037591	-	0.002372
XLOC_037597	-	0.008746
XLOC_037604	-	0.032252
XLOC_037626	-	0.026101
XLOC_037634	-	0.002372
XLOC_037636	-	0.025115
XLOC_037661	-	0.002372
XLOC_037667	-	0.005845
XLOC_037672	-	0.002372
XLOC_037763	-	0.013835
XLOC_037767	-	0.012580
XLOC_037795	-	0.019332
XLOC_037821	-	0.019332
XLOC_037827	-	0.004177
XLOC_037882	-	0.002372
XLOC_037889	-	0.002372
XLOC_038028	-	0.031419
XLOC_038085	-	0.004177
XLOC_038103	NR4A1	0.002372
XLOC_038122	ENSSSCG00000004124	0.031419
XLOC_038225	-	0.011350

XLOC_038265	-	0.002372
XLOC_038283	ALS2	0.031419
XLOC_038322	-	0.002372
XLOC_038362	SEMA7A	0.002372
XLOC_038364	-	0.002372
XLOC_038369	-	0.038059
XLOC_038373	-	0.002372
XLOC_038546	-	0.030613
XLOC_038549	ENSSSCG00000021712	0.039463
XLOC_038669	ENSSSCG00000002453	0.036488
XLOC_038672	DAGLB	0.002372
XLOC_038685	ENSSSCG00000021949	0.002372
XLOC_038714	ENSSSCG00000028099	0.002372
XLOC_038767	ANG1,RSE4	0.002372
XLOC_038772	-	0.017207
XLOC_038773	-	0.047338
XLOC_038782	-	0.002372
XLOC_038783	-	0.034852
XLOC_038815	-	0.005845
XLOC_038823	ENSSSCG00000026670	0.038749
XLOC_038828	ENSSSCG00000025390	0.002372
XLOC_038892	-	0.024202
XLOC_038902	CPNE2	0.002372
XLOC_038921	-	0.010072
XLOC_038929	MCTP2	0.048740
XLOC_038931	-	0.044653
XLOC_038983	-	0.002372
XLOC_038984	-	0.002372
XLOC_038985	-	0.002372
XLOC_039011	-	0.002372
XLOC_039051	ASAP3	0.002372
XLOC_039081	-	0.012580
XLOC_039085	-	0.002372
XLOC_039086	-	0.002372
XLOC_039087	-	0.002372
XLOC_039089	-	0.038059
XLOC_039109	GZMM	0.002372
XLOC_039441	-	0.002372
XLOC_039560	-	0.023280
XLOC_039778	ETV5	0.024202
XLOC_039847	-	0.005845
XLOC_039864	-	0.041076
XLOC_039952	-	0.039463
XLOC_039995	-	0.002372

XLOC_040288	-	0.004177
XLOC_040301	ENSSSCG00000024533	0.012580
XLOC_040309	ENSSSCG00000022360	0.002372
XLOC_040310	-	0.004177
XLOC_040315	-	0.002372
XLOC_040317	-	0.036488
XLOC_040352	ENSSSCG00000022971	0.012580
XLOC_040361	-	0.030613
XLOC_040392	-	0.032252
XLOC_040497	RSE6	0.002372
XLOC_040581	CYFIP1	0.002372
XLOC_040599	-	0.002372
XLOC_040612	-	0.002372
XLOC_040619	IRF8	0.038059
XLOC_040688	ETS2	0.002372
XLOC_040694	-	0.017207
XLOC_040847	ENSSSCG00000027764	0.047338
XLOC_040920	-	0.018305
XLOC_040928	-	0.005845
XLOC_040956	-	0.023280
XLOC_040957	HMOX1	0.002372
XLOC_040967	-	0.002372
XLOC_040968	UPP1	0.017207
XLOC_040978	SLC2A5	0.026101
XLOC_040979	-	0.004177
XLOC_041039	S100A11	0.008746
XLOC_041074	-	0.037305
XLOC_041094	ENSSSCG00000024640	0.045290
XLOC_041120	-	0.008746
XLOC_041169	-	0.002372
XLOC_041183	-	0.031419
XLOC_041194	-	0.013835
XLOC_041241	-	0.029810
XLOC_041250	-	0.025115
XLOC_041341	-	0.037305
XLOC_041345	-	0.017207
XLOC_041346	-	0.018305
XLOC_041347	-	0.002372
XLOC_041348	-	0.030613
XLOC_041350	-	0.010072
XLOC_041413	-	0.007395
XLOC_041480	-	0.004177
XLOC_041501	ENSSSCG00000026336	0.002372
XLOC_041524	-	0.002372

XLOC_041525	-	0.002372
XLOC_041526	-	0.002372
XLOC_041528	-	0.002372
XLOC_041529	-	0.002372
XLOC_041530	-	0.033995
XLOC_041532	-	0.018305
XLOC_041533	-	0.012580
XLOC_041534	-	0.007395
XLOC_041535	-	0.024202
XLOC_041536	-	0.044653
XLOC_041613	SLC37A3	0.028943
XLOC_041626	-	0.034852
XLOC_041657	IFR2	0.011350
XLOC_041668	HIATL1	0.002372
XLOC_041675	DCTN6	0.048740
XLOC_041718	AKR1A1	0.042609
XLOC_041737	-	0.002372
XLOC_041774	-	0.013835
XLOC_041775	-	0.007395
XLOC_041776	-	0.010072
XLOC_041789	TRIB1	0.047338
XLOC_041875	-	0.019332
XLOC_041911	ENSSSCG00000027269	0.002372
XLOC_041939	-	0.004177
XLOC_041950	-	0.008746
XLOC_041962	ENSSSCG00000021402	0.002372
XLOC_042002	CSF3R	0.002372
XLOC_042011	WDR73	0.004177
XLOC_042021	ISG15	0.008746
XLOC_042052	ENSSSCG00000021766	0.015000
XLOC_042089	-	0.002372
XLOC_042120	-	0.033125
XLOC_042124	-	0.018305
XLOC_042126	SEPX1	0.012580
XLOC_042224	ENSSSCG00000026820	0.002372
XLOC_042246	-	0.049392
XLOC_042247	RNH1	0.038749
XLOC_042274	-	0.002372
XLOC_042288	ENSSSCG00000028609	0.048740
XLOC_042308	CECR1	0.002372
XLOC_042316	EMR4	0.002372
XLOC_042323	S100B	0.002372
XLOC_042384	ENSSSCG00000028437	0.013835
XLOC_042494	PDXK	0.002372

XLOC_042590	CSF2RA	0.002372
XLOC_042599	PYGB	0.045889
XLOC_042600	ABHD12	0.002372
XLOC_042621	-	0.041076
XLOC_042638	ARHGEF40	0.025115
XLOC_042713	C3AR1	0.002372
XLOC_042724	TNFRSF1B	0.007395
XLOC_042756	SNORD107	0.046618
XLOC_042757	SNRPN	0.004177
XLOC_042819	PRSS57	0.002372
XLOC_042822	-	0.002372
XLOC_042846	ENSSSCG00000021006	0.008746
XLOC_042848	-	0.010072
XLOC_042936	-	0.002372
XLOC_042937	CDKN1B	0.002372
XLOC_042939	-	0.012580
XLOC_042951	-	0.026101
XLOC_042952	-	0.035659
XLOC_042967	DPP7	0.002372
XLOC_043046	-	0.002372
XLOC_043048	-	0.002372
XLOC_043098	-	0.024202
XLOC_043099	-	0.041076
XLOC_043128	PTGER4	0.005845
XLOC_043147	S100A6	0.002372
XLOC_043150	KCNJ2	0.002372
XLOC_043155	-	0.002372
XLOC_043167	-	0.047338
XLOC_043168	-	0.002372
XLOC_043169	-	0.016103
XLOC_043210	ENSSSCG00000001071	0.004177
XLOC_043228	ENSSSCG00000016503	0.025115
XLOC_043264	-	0.023280
XLOC_043301	MAP3K11	0.012580
XLOC_043303	-	0.002372
XLOC_043402	NRP2	0.002372
XLOC_043422	-	0.002372
XLOC_043426	ENSSSCG00000025114	0.033995
XLOC_043483	ENSSSCG00000011117,ENSSSCG00000029195	0.019332
XLOC_043484	COMMD6	0.041848
XLOC_043491	ABHD10	0.002372
XLOC_043495	DSC2	0.002372
XLOC_043657	ENSSSCG00000022138	0.036488

XLOC_043659	ENSSSCG00000023735	0.002372
XLOC_043663	ENSSSCG00000030268	0.039463
XLOC_043667	GPRC5C	0.015000
XLOC_043668	-	0.023280
XLOC_043669	-	0.002372
XLOC_043717	TLR4	0.023280
XLOC_043955	-	0.024202
XLOC_043964	ENSSSCG00000028219	0.044005
XLOC_043981	ENSSSCG00000022587	0.027058
XLOC_043987	-	0.004177
XLOC_043989	-	0.002372
XLOC_043992	-	0.043224
XLOC_043995	-	0.002372
XLOC_043997	-	0.002372
XLOC_043998	-	0.012580
XLOC_044001	-	0.020320
XLOC_044002	-	0.029810
XLOC_044004	-	0.004177
XLOC_044005	-	0.043224
XLOC_044044	-	0.031419
XLOC_044284	TBL1Y	0.042609
XLOC_044290	WWC3	0.007395
XLOC_044313	BMX	0.008746
XLOC_044340	PRDX4	0.045290
XLOC_044370	GK	0.002372
XLOC_044394	CYBB,XK	0.005845
XLOC_044401	ENSSSCG00000012238	0.010072
XLOC_044404	CH242-236H6.1	0.004177
XLOC_044406	ATP6AP2	0.002372
XLOC_044426	RP2	0.002372
XLOC_044433	CFP,CH242-69I19.6,ELK1,UXT	0.002372
XLOC_044438	ENSSSCG00000021591	0.002372
XLOC_044461	CLCN5	0.002372
XLOC_044501	ENSSSCG00000012359	0.048079
XLOC_044506	-	0.002372
XLOC_044508	VSIG4	0.002372
XLOC_044517	PJA1	0.026101
XLOC_044572	ENSSSCG00000012439	0.002372
XLOC_044577	P2Y10	0.004177
XLOC_044582	-	0.002372
XLOC_044627	P1L3	0.038749
XLOC_044641	BHLHB9,GPRASP2	0.002372
XLOC_044661	TBC1D8B	0.002372
XLOC_044718	-	0.031419

XLOC_044735	SH2D1A	0.002372
XLOC_044742	OCRL	0.012580
XLOC_044759	-	0.011350
XLOC_044861	-	0.007395
XLOC_044862	MPP1	0.002372
XLOC_044867	G6PD	0.008746
XLOC_044998	CASK	0.038059
XLOC_045004	MAOA	0.005845
XLOC_045005	MAOB	0.002372
XLOC_045013	ssc-mir-221,ssc-mir-222	0.002372
XLOC_045025	TIMP1	0.002372
XLOC_045097	ARHGEF9	0.030613
XLOC_045103	ENSSSCG00000028241	0.002372
XLOC_045152	PGK1	0.005845
XLOC_045155	GPR174	0.002372
XLOC_045254	ACSL4,CH242-17O13.4,KCNE5	0.044653
XLOC_045284	SLC25A43	0.015000
XLOC_045287	LONRF3	0.002372
XLOC_045290	IL13RA1	0.008746
XLOC_045292	KLHL13	0.021314
XLOC_045396	RENBP	0.002372
XLOC_045420	-	0.005845
XLOC_045436	-	0.002372
XLOC_045474	-	0.002372
XLOC_045476	-	0.002372
XLOC_045483	-	0.026101
XLOC_045485	-	0.002372
XLOC_045496	-	0.002372
XLOC_045522	-	0.002372
XLOC_045563	-	0.038749
XLOC_045570	-	0.002372
XLOC_045575	-	0.002372
XLOC_045710	-	0.002372

Supplementary Table 3. Product sizes and the primers used for qRT-PCR

Gene	GenBank accession Number	Forward Primer(5'-3')	Reverse Primer(5'-3')	Amplicon Size (bp)
B2M	ENSSSCG00000004682	GCGGAAAACGGAAAGCCAAA	CACGCGGCAGCTATACTGAT	201
PTI	ENSSSCG00000025317	TGGCTTTAGTAGCCCTGGATG	GCATTTTGCTCGGTGGTGTT	189
C3AR1	ENSSSCG00000023868	TGCCCTCCTGGGGAAAGATA	ACTGGGTGGAGCCCTAAGAA	200
CCL5	ENSSSCG00000017705	GAAATGGGTGCGGGAGTACA	AGGAGCCCTGGGAGGTTTTA	215
CCL8	ENSSSCG00000017721	GATCACCTGCTGCTTCGGT	TCAAGGCTTTGGGGTTTGGG	208
CCR1	ENSSSCG00000011322	CCCAGTGGGAGTTCAGTCAC	ATCAGACGCACAGCTTTGGA	202
CCRL2	ENSSSCG00000023557	CCTCAACCTGGCAGTGTGTA	TGGCCGAGAAAAACCCTCTC	212
GZMA	ENSSSCG00000016903	ATGTGTGTCGAGTTGCAGGAT	TGCAGGAGTCTTTTCCACCTT	189
GZMH	ENSSSCG00000022894	ACTGCAGGGGAAGCTCAATC	ACAGCCTTAGTCAGCTTGGC	181
P2RY13	ENSSSCG00000011716	CTGGGGCTGAAATGGCATCA	AGAAGACAGCCACGACAACAA	193
TFPI	ENSSSCG00000016032	GAACCCCTCTTGAGGCTTTGA	ACTGTACTTAAAGGGGCGGC	185
TLR4	ENSSSCG00000005503	AGCTGTATCGCCTTCTCAGC	ACGTTGGGAGTTTTCCCTCC	193
TLR6	ENSSSCG00000026592	CTGCCTGGATGTGGTTCCTT	CAGGCCAACTCTCTACCACG	208

Supplement Table 4.Gene fold change tested by qRT-PCR and RNA-Seq

Gene	qRT-PCR								RNA-Seq							
	Failure-1 / Baseline-1	Failure-2 / Baseline-2	Failure-3 / Baseline-3	Failure-4 / Baseline-4	Dying-1/ Baseline-1	Dying-2 / Baseline-2	Dying-3 / Baseline-3	Dying-4 / Baseline-4	Failure-1 / Baseline-1	Failure-2/ Baseline-2	Failure-3 / Baseline-3	Failure-4 / Baseline-4	Dying-1 / Baseline-1	Dying-2 / Baseline-2	Dying-3 / Baseline-3	Dying-4 / Baseline-4
PTI	1.0	2.2	1.9	2.5	0.5	1.7	1.3	0.5	1.3	3.6	1.9	2.0	0.8	2.5	1.5	0.5
C3AR1	11.4	11.1	4.9	15.0	8.7	13.7	12.3	19.8	3.4	7.5	5.1	11.7	4.3	10.7	6.5	13.5
CCL5	0.2	0.3	0.2	0.5	0.4	0.2	0.2	0.6	0.5	0.2	0.3	0.4	0.8	0.2	0.4	0.4
CCL8	29.7	3.8	9.6	2.1	30.5	6.5	63.5	29.8	19.6	5.5	10.1	1.3	20.0	7.6	48.6	16.8
CCR1	3.5	2.2	2.1	6.3	2.0	2.1	2.7	3.6	1.5	3.7	2.6	5.4	1.0	3.4	1.8	4.3
CCRL2	2.9	1.0	1.9	2.5	2.5	1.6	3.1	4.9	2.1	1.6	1.9	2.0	1.8	1.9	3.1	4.2
GZMA	5.8	0.2	0.3	0.7	8.5	0.1	0.2	0.6	0.6	0.2	0.3	0.8	0.8	0.1	0.3	0.6
GZMH	0.6	0.2	0.3	0.4	0.6	0.1	0.3	0.5	0.6	0.2	0.3	0.2	0.5	0.1	0.4	0.3
P2RY13	3.4	3.1	2.9	1.3	1.3	2.2	2.4	3.6	9.6	5.4	3.5	2.1	6.1	5.2	4.2	7.2
TFPI	1.9	2.9	1.5	2.6	0.7	1.4	1.1	0.3	1.3	5.0	2.1	3.7	0.7	2.3	1.3	0.4
TLR4	2.3	2.9	1.4	4.2	1.3	6.9	2.7	6.2	1.1	1.8	1.4	4.1	1.6	4.0	2.2	5.4
TLR6	4.5	1.2	3.4	1.7	1.8	1.4	10.3	7.9	1.1	1.4	2.0	1.6	1.5	1.9	2.1	2.1
